# Microplastics from Food Packaging: Polymer Degradation Pathways, Environmental Distribution, and Effects on the Human Gastrointestinal Tract

**DOI:** 10.3390/polym17212923

**Published:** 2025-10-31

**Authors:** Monika Lewanska, Renata Barczynska

**Affiliations:** Department of Dietetics and Food Studies, Faculty of Science and Technology, Jan Dlugosz University in Czestochowa, Al. Armii Krajowej 13/15, 42-200 Czestochowa, Poland; m.lewanska@ujd.edu.pl

**Keywords:** microplastics, food packaging, polymer, environment pollution, degradation, food safety, gastrointestinal tract, human health

## Abstract

Fossil fuels are currently the primary source for plastic production, with global production exceeding 400 million tons annually. The food sector remains the dominant application, particularly in the production of single-use packaging. Commonly used packaging is primarily made from PE, PP, PS, and PET. The versatility of these materials stems from their lightweight, functionality, and ability to extend the shelf life of food products. Unfortunately, constantly growing consumption generates vast amounts of difficult-to-degrade waste, which in the natural environment constantly fragments, generating hazardous microplastics (MPs). MPs readily spread throughout the biosphere and are now commonly detected in the digestive tracts of humans and animals. Current scientific reports indicate their potential contribution to the pathogenesis of numerous diseases such as inflammatory bowel disease, diabetes, obesity, allergic reactions, and cancer. This link is believed to result from mechanisms involving physical toxicity, exposure to chemical substances, and microbiological interactions. The MP problem is global in nature and encompasses the entire life cycle of plastics, from production to accumulation in living organisms. This review aims to provide an up-to-date and comprehensive overview of the toxicological and environmental issues related to MPs, addressing the current research gaps and emphasizing their increasing relevance to human health.

## 1. Introduction

Due to their excellent performance properties and versatile practicality, plastics have gained enormous popularity, with global production of these materials already exceeding 400 million tons. The main raw materials used in the production of plastics are fossil fuels (crude oil, natural gas, and coal) [1], 50% of which serve as raw materials, and the remaining 50% are used as fuel in the conversion process. One of the main consumers of plastics is the food industry, and their consumption is primarily associated with the production of packaging and other disposable materials. Thermoplastics (84%) are most commonly used to produce disposable packaging materials (SUP) used in the food industry, including polyethylene (PE), polypropylene (PP), polystyrene (PS), and polyethylene terephthalate (PET) [2,3]. Globally, 60% of all plastic packaging is used strictly for food and beverage packaging. It is estimated that we buy 1 million plastic bottles per minute, and 5 trillion plastic bags are used globally each year [4]. Packaging is a key element of the production and distribution chain and plays a crucial role for consumers. It fulfills many key functions, including protecting products from damage, mechanical stress, and contamination during transport and storage, preserving their properties and shelf life, and serving presentation, esthetic, and informational purposes [5]. Packaging also contributes to lower transportation costs in the food sector. Transporting food and beverages in glass containers is more expensive due to the heavier weight of glass compared to lightweight plastic. Therefore, the lighter weight of plastic packaging allows for the transport of larger quantities of products at lower costs [4,6]. Food packaging has enabled the dynamic development of the food industry that serves fast food, ready-made meals, snacks, and take-out beverages. The demand for food is growing every year as the world’s population grows, which is estimated to reach 9.7 billion by 2050. This demand for food will also increase significantly, and consequently, the production of packaging materials will also increase. Once it has served its purpose, unnecessary packaging and disposable products are discarded and become waste. The plastics industry has been generating enormous amounts of waste since the 1950s, and this amount is increasing every year. Every day, a large portion of mixed plastic waste, which is difficult to manage, is released into the environment without any supervision [2]. Others end up in landfills, incinerators, or ultimately recycling plants. Packaging waste can persist in the environment for a very long time, largely determined by the type of plastic and the conditions in which it is stored [2]. Plastic materials are extremely useful due to their versatile properties. However, the risks associated with their mass production were not fully recognized [7]. MPs are irregular fragments of plastics, smaller than 5 mm, resulting from the unintentional degradation of larger components. MPs are released into the environment primarily as a result of atmospheric conditions and microbiological activity, and their size determines their easy dissemination. Therefore, it was only a matter of time before their fragments were detected in the gastrointestinal tract [8,9].

Studies confirm the presence of MP fragments in every part of the digestive system. Although there is currently no clear evidence linking the consumption of plastic particles to human health, the results of experiments on animal models, correlational studies with individuals exposed to high levels of MPs, and observations of cell lines suggest that the effects of MP exposure include numerous reactions in the gastrointestinal tract correlated with the development of diseases such as intestinal inflammation, obesity, diabetes, allergic reactions, circulatory diseases, autoimmune diseases, and cancer [10]. Humans are likely to suffer health consequences from contact with MPs directly due to their physical presence (abrasion leading to cytotoxicity, inflammation, oxidative stress), chemical exposure (monomers, production additives, adsorbed contaminants causing systemic toxicity or an immune response), or microbial exposure (carriers of dangerous, often drug-resistant, pathogens causing infections or intestinal dysbiosis). Evidence that humans consume MPs is provided by the presence of MPs in human feces. PP and PET fragments are the most commonly found. A positive correlation has been reported between MP concentration in feces and the severity of inflammatory bowel disease (41.8 items/g in patients with inflammatory bowel disease, 28 items/g in healthy individuals) [11]. This review addresses the issue of MPs from their source to the environment and the human body. It is a complex and multi-stage process that begins at the production stage, progressing through use, further degradation, and distribution. MPs are now recognized as a global environmental contaminant, and although it is widely recognized that humans are exposed to MPs throughout their lives, issues regarding their variability, concentration, accumulation, and even exposure and consumption rates worldwide remain unresolved [12]. Considering the widespread use of plastics, the increasing production of single-use packaging, and the emerging evidence of MPs contamination in the environment and the human body, as well as ongoing degradation processes that continuously generate persistent MPs and increase their abundance in the environment, this review is timely and needed to synthesize current knowledge, highlight research gaps, and provide insights into the potential health risks associated with MPs.

## 2. Plastics Applied in the Food Industry

**Figure 1:**
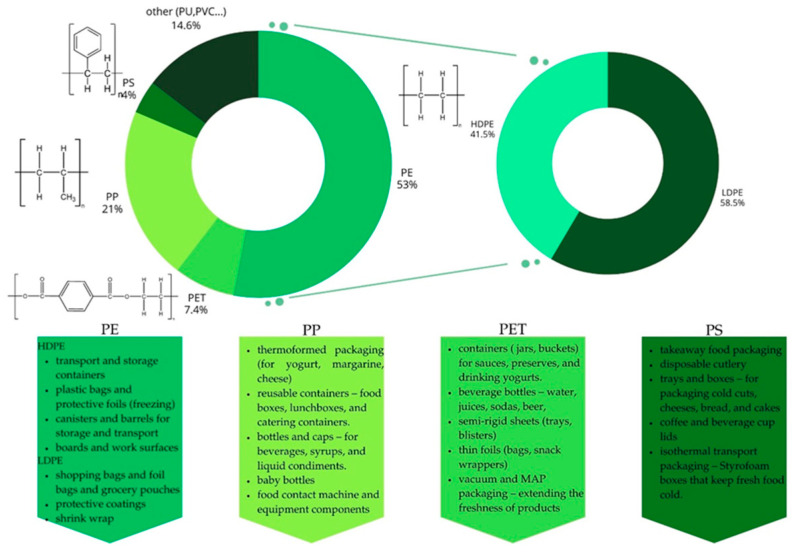
The most commonly used plastics in packaging are low-density polyethylene (LDPE), which together account for 31% of total consumption. Next are high-density polyethylene (HDPE) at 22%, polypropylene (PP) at 21%, polyethylene terephthalate (PET) at 7.4%, and polystyrene (PS) at 4%. In the food industry, these plastics are used to manufacture various types of food-contact packaging.

Plastics in the food industry are primarily used as disposable food packaging materials. They have gained popularity mainly due to their low production costs, lightweight nature, and resistance to physical, chemical, and biological factors [13]. In recent years, the processing properties of plastics have been significantly improved, thus increasing their application potential. Improvements have been achieved by adding additives to classic plastics, such as dyes, plasticizers, stabilizers, fillers, colorants, reinforcing fibers, antioxidants, ultraviolet absorbers, and other compounds that aid in processing [13]. Plastics can take on various shapes and properties by changing their chemical structure. Polymeric materials are used as base materials for the production of flexible (83%) and rigid (45%) packaging, as well as supplementary materials in multilayer or multimaterial packaging [3]. Precise classification of plastics is difficult due to the wide variety of materials. Everything depends on criteria such as structure, origin, application, physicochemical properties, and source [13]. Taking into account the different types of polymerization, plastics can be divided into two groups. The first group includes polymers formed by carbon backbones (C–C) linked by sigma bonds, where the double carbon bonds present in the monomers form new carbon bonds (C–C) through condensation reactions. This group includes PE, PP, PS, and PVC, which account for 77% of the market share. The second group comprises polymers containing a backbone containing other elements, resulting from the condensation reaction of hydroxyl and carboxyl or amine groups, leading to the formation of ester bonds (polyesters) or amide bonds (polyamides). This group includes PET and PU, which account for 23% of the market share [1]. Based on their thermal properties, plastics can be divided into thermoplastics and thermosets. Thermoplastics are polymers that do not undergo permanent chemical changes when heated, allowing them to be repeatedly melted and molded without losing their functional properties. The presence of a stable carbon backbone in the structure of thermoplastics determines their resistance to degradation processes, particularly hydrolysis. These include acrylonitrile-butadiene-styrene (ABS), polyethylene (PE), polyamide (PA), polyethylene terephthalate (PET), polyimide (PI), polypropylene (PP), polymethyl methacrylate (PMMA), polystyrene (PS), polytetrafluoroethylene (PTFE), polyvinyl chloride (PVC), and polyvinylidene chloride (PVDC). Thermosets, unlike thermoplastics, cannot be recycled by melting because they undergo irreversible chemical reactions at high temperatures. These materials are characterized by a highly cross-linked, heteroatomic structure, which makes them more susceptible to hydrolytic cleavage. These include acrylic and epoxy resins, silicone and vinyl resins, and polyurethane (PU) [14]. In the food sector, the most commonly used plastics include PE, PP, PS, and PET (Figure 1).

### 2.1. Polyethylene

PE is a thermoplastic resin produced by the polymerization of ethylene. The material exhibits different properties depending on its molecular structure and density [15]. PE is the leading material in the packaging market, accounting for 40% of global production of non-fiber polymer resins (2019) [6]. PE was first synthesized in 1898, and the discovery of metal catalysts enabled polymerization under much milder conditions [16]. The method of preparation allows for distinguishing between two basic types of polyethylene. The high-pressure process produces LDPE, i.e., low-density polyethylene (ρ: 0.910–0.925 g/cm^3^, branching density 20–40/1000C), while the low-pressure process allows the production of HDPE, i.e., high-density polyethylene (ρ > 0.941 g/cm^3^; branching density <10/1000C) [17]. There are two types of LDPE: linear low-density polyethylene (LLDPE) and branched low-density polyethylene (BLDPE). In addition to the degree of branching, both polymers differ in density and the availability of surface functional groups. LDPE is widely used for food packaging and in the production of trays and bags. It is also used to coat products made from other materials, such as paper, textiles, and other plastics. Films made from this material are transparent, odorless, highly malleable, heat-sealable, and have low water vapor permeability. LDPE has a crystallinity of 50–60%, making it resistant to stretching and tearing, chemically resistant, and flexible even at low temperatures. HDPE has few branches, resulting in stronger intermolecular interactions and better resistance to stretching. Its high density allows for good stability, hardness, resistance, and durability at high temperatures up to 120 °C. All these characteristics have allowed it to be widely used in everyday life. It is used to produce transport bags, packaging, bottles, and food containers [18]. In North America, in 2021, it accounted for 42% of plastic resin sales by weight, with 68% used in packaging production. It is estimated (OECD) that by 2060, the quantities of low-density polyethylene (LDPE) and linear low-density polyethylene (LLDPE) for food applications will triple compared to 2019 [6].

### 2.2. Polypropylene

PP is an economical thermoplastic polymer characterized by favorable properties such as flame retardance, ease of processing, high heat distortion temperature, and dimensional stability [19]. The versatility of this material stems from its unique molecular arrangement, which contains monomers linked together in a straight chain, and the linear configuration offers several advantages, including increased crystallinity, better chemical resistance, lower density, and satisfactory mechanical strength [20]. PP can occur in three different structural forms: atactic, isotactic, and syndiotactic. The type of structure depends on the arrangement of the pendant methyl groups along the main polymer chain [21]. PP was accidentally discovered in 1951 and quickly found widespread use due to having the lowest density among commonly used polymers [22]. It currently accounts for 16% of the global plastics market [19]. Propylene polymerization occurs via a coordination mechanism involving a catalyst. Catalysts are a key element of production technology and a major driver of this industry’s development. They directly influence the final product’s properties, such as molecular weight, morphology, isotacticity, and particle size distribution [22]. PP quality in industrial practice is conventionally assessed based on the melt flow index (MFI) [23]. Based on its physicochemical properties, polypropylene (PP) is divided into three main types: homopolymer (HPP), random copolymer (RCP), and impact copolymer (ICP). PP homopolymer (HPP) consists exclusively of propylene monomers and occurs in a semi-crystalline form. PP random copolymer (RCP) contains ethylene admixtures (1–8%), which improve its flexibility and transparency. Impact copolymer PP, on the other hand, is a variant of HPP to which a co-mixed RCP phase ethylene has been added, significantly improving the material’s impact strength, especially at low temperatures. Of these three types, HPP is the most commonly used type of polypropylene in the plastics industry [19]. PP is widely used in applications ranging from disposable products to durable, long-life items, thanks to its excellent properties and competitive price. One of PP’s initial limitations was its low impact strength, especially at low temperatures, but this has been significantly improved thanks to PP’s excellent compatibility with polyolefin rubbers, which has broadened its range of applications. PP has a melting point of approximately 160 °C, which allows for the sterilization of packaging containing sensitive ingredients after filling, thus extending the product’s shelf life. An additional advantage is the ability to monitor the freshness of packaged products thanks to the increased transparency of PP films, achieved through optimized processing conditions. As a result, the amount of polypropylene waste is increasing, as this material is widely used in the production of a variety of disposable packaging [24].

### 2.3. Polystyrene

PS is a synthetic polymer obtained from monomer units of styrene, a liquid hydrocarbon derived from the petrochemical industry. It was first developed by the German company BASF and is currently widely used in various engineering fields [25]. Over 25 million tons of PS are produced worldwide annually, representing approximately 6% of total plastics production [26]. A 4% average annual production growth is forecast for 2021–2026, driven by growing demand for this material, particularly in the packaging sector, including food packaging. Polystyrene is widely used in food packaging because it provides good thermal insulation but is permeable to water vapor and gases. These properties make it ideal for packaging meat and products with a short shelf life. PS is produced by polymerizing styrene, which involves heating in the absence of air [25]. Based on its properties, PS is divided into three main types: GPPS, HIPS, and EPS. GPPS (General Purpose Polystyrene) is the most commonly used form of polystyrene. It is characterized by stiffness, high transparency, a glossy surface, and low water permeability. It has a solid structure, is resistant to gamma radiation, and can withstand acidic and alkaline environments. However, its disadvantages include low resistance to chemicals and harsh environmental conditions. GPPS is mainly used for producing disposable cups and food containers. However, it is not recommended for contact with high-fat foods because it can be subject to stress cracking, which weakens its barrier properties. HIPS (High Impact Polystyrene) is a variety of PS designed to counteract the brittleness of GPPS. It contains synthetic rubbers that increase its impact resistance and flexibility. Unlike GPPS, HIPS is opaque, less glossy, and has lower structural stability and higher permeability. It also exhibits lower resistance to gamma radiation and chemical resistance, although it better withstands environmental stresses. It may interact with substances present in food, such as ethanol, n-heptane, or isooctane, which can lead to their migration into the food product. EPS (Expandable Polystyrene) is an expandable type of PS consisting of microgranules, which are foamed using pentane. Thanks to its properties—high impact resistance, very good thermal insulation, and ease of processing—EPS is widely used as a thermal insulation material [25].

### 2.4. Polyethylene Terephthalate

PET is one of the most important plastics produced worldwide due to its high durability, excellent mechanical properties, transparency, non-toxicity, recyclability, and good processability. Furthermore, this material is characterized by a favorable balance between functional properties and low production costs [27]. Currently, global production of PET-based plastics is approximately 70 million tons per year, which accounts for approximately 12% of the total mass of municipal solid waste worldwide [28]. Due to its lightness, durability, and versatility, PET is a widely used polyester essential in the food sector, mainly for the production of packaging products such as bottles for water and other beverages (carbonated drinks, beer, juice, milk), food containers, trays, films, sheets, and vacuum packaging [29,30]. Since its introduction in 1953 as a cheap and efficient textile fiber, PET has become the most widely used and produced fiber-forming polymer, accounting for approximately 90% of all synthetic fibers [31]. The PET monomer contains an aromatic ring linked to a short aliphatic chain, which gives the molecule considerable stiffness compared to fully aliphatic polymers such as polyolefins or polyamides [32]. PET is a semi-crystalline polymer because it consists of both highly ordered crystalline regions and disordered amorphous regions [33]. Commercial PET is obtained by polycondensation of ethylene glycol (EG) with terephthalic acid (TPA). On an industrial scale, EG and TPA (or DMT) are produced from ethylene and p-xylene, respectively. Currently, the dominant method of PET synthesis is the catalytic conversion of ethylene to ethylene oxide. It is worth noting that the dominant raw materials for ethylene and p-xylene production come from non-renewable fossil sources, such as crude oil, coal, and natural gas. Due to the high risk of greenhouse gas emissions and the depletion of fossil fuel resources, increasing attention is being paid to developing technologies for producing PET monomers from renewable sources, e.g., through the transformation of biomass [31].

## 3. Polymer Degradation

**Figure 2:**
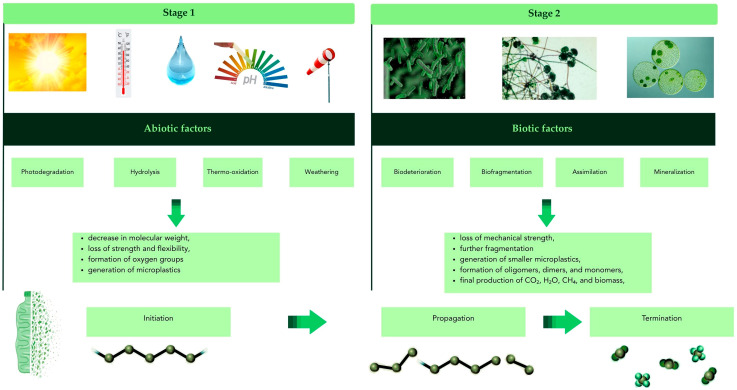
Degradation of polymers in the environment. The influence of abiotic and biotic factors on the structure of polymer materials.

Plastic degradation processes depend on polymer chain length, additives used in production, generated radicals, and environmental conditions [12]. The environmental impact of each plastic is determined by the stages of its life cycle, starting with raw material extraction, through production, transport, distribution, and use, and ending with disposal [6]. Broadly speaking, polymer degradation depends on abiotic and biotic exposure conditions (Figure 2). Geographical location, weathering, air pollution, and climate can also influence the mechanisms and rate of the degradation process [34,35,36]. In nature, degradation begins with photodegradation, followed by hydrolysis and thermooxidation. Degradation occurs due to the action of various abiotic factors such as UV light (it is reported that the intensity of sunlight is the main determining factor, and the rate of the degradation process increases with the intensity of sunlight because the rate of photooxidation increases [37]), temperature (the rate of polymer degradation increases with rising temperature. Higher temperatures also increase the mobility of polymer chains, which in turn can promote greater substrate availability and intensify enzymatic activity [38]. Humidity enables the hydrolytic cleavage of functional groups. This leads to fragmentation of polymer chains, which accelerates further stages of degradation [37]. Water can react with atoms of elements such as oxygen, nitrogen, sulfur, phosphorus, and other non-carbon elements. The surrounding carbon atoms are positively charged due to the electron-withdrawing properties of these elements. When water is near these polymers, the oxygen atom in the water molecule attacks the positively charged carbon atom, which serves as the nucleophilic polymer core [26], salinity, and pH [37]. Biotic degradation significantly impacts the mechanical strength and hydrophobicity of the material, alters its appearance, and increases brittleness. These changes, occurring at the molecular level, lead to a loss of mechanical properties, which in turn facilitates fragmentation and the generation of smaller micro- and nanoplastics. These processes are responsible for the breakdown of waste polymers into compounds with even smaller molecular weights (oligomers, dimers, monomers), which can then be metabolized by microorganisms (biotic factors) [39]. Plastic biodegradation involves a series of biochemical steps that can be classified as: biodeterioration, biofragmentation, assimilation, and mineralization. Each of these processes involves specific enzymatic activities and leads to the gradual cleavage of chemical bonds in the polymer structure [14]. Microorganisms such as bacteria, fungi, and algae can degrade polymers through their metabolic processes under aerobic or anaerobic conditions without consuming heat or generating harmful byproducts [37].

Biotic degradation significantly impacts the mechanical strength and hydrophobicity of the material, alters its appearance, and increases brittleness. These changes, occurring at the molecular level, lead to a loss of mechanical properties, which in turn facilitates fragmentation and the generation of smaller micro- and nanoplastics. These processes are responsible for the breakdown of waste polymers into compounds with even smaller molecular weights (oligomers, dimers, monomers), which can then be metabolized by microorganisms (biotic factors) (Table 1) [39]. Plastic biodegradation involves a series of biochemical steps that can be classified as: biodeterioration, biofragmentation, assimilation, and mineralization. Each of these processes involves specific enzymatic activities and leads to the gradual cleavage of chemical bonds in the polymer structure [14]. Microorganisms such as bacteria, fungi, and algae can degrade polymers through their metabolic processes under aerobic or anaerobic conditions without releasing heat or producing harmful byproducts [37]. It should also be remembered that plastics have only been present in the environment for decades, so targeted enzymatic systems for the microbial degradation of individual polymers may not have evolved yet [40]. The first mentions of the destructive effects of microorganisms on polymers date back to the first half of the 20th century. Biodegradation here refers to mechanisms that lead to the destruction of organic substances by living organisms. In the case of high-molecular-weight polymers, degradation to CO_2_ requires the cooperation of multiple consortia of microorganisms with diverse metabolic properties. This results in the gradual decomposition of the plastic into smaller fragments, down to monomers, which in turn can be further processed by other types of microorganisms into simple compounds, ultimately degraded by yet another group of microbes. The ability to degrade a polymer depends on the presence of special intracellular or extracellular depolymerases, operating under appropriate environmental conditions. Enzymes secreted by microorganisms catalyze degradation reactions primarily at distal polymer fragments. Among the microorganisms characterized by the ability to biodegrade plastics are microbes isolated from various environments (water, soil, compost, landfills), i.e., those where polymer contamination is common [41].

Fungi play a key role in the degradation of polymeric materials. Their mycelium can effectively penetrate the surface of the material and extend into deeper layers, enabling more effective degradation of the substrate. Additionally, fungi secrete extracellular depolymerases, which break down polymers into smaller fragments—oligomers, dimers, and monomers. The resulting monomers can then be taken up by fungal cells, where they are further assimilated or mineralized by intracellular enzymatic systems [65]. Compared to bacteria, fungi produce and secrete significantly larger amounts of enzymes, making them particularly effective in the degradation of polymeric materials. In this context, white and brown rot fungi are often cited as exceptionally efficient degraders. Their ability to degrade polymeric materials stems from an active enzymatic system, which includes, among others, manganese peroxidase (MnP), lignin peroxidase (LiP), versatile peroxidase, and laccase (Lac). These enzymes are capable of effectively degrading and even mineralizing lignin, which also translates into their ability to degrade other complex organic compounds. Polymeric materials share similarities with lignin in both their physical properties and chemical structure. Features such as hydrophobicity and the presence of ether bonds, non-phenolic aromatic rings, and a carbon chemical skeleton resemble the structure of lignin, which undergoes oxidation during its degradation. These physicochemical similarities enable the degradation of some plastics, such as polyethylene (PE) or polypropylene (PP), with the participation of lignin-modifying enzymes (LMEs) such as MnP or Lac [34]. Many bacterial strains also demonstrate high efficiency in the degradation of plastics, with particularly promising results obtained under conditions of interaction between multiple microbial species. Nevertheless, in most cases, the rate of polymer biodegradation by fungi exceeds that of bacteria. A clear advantage of bacteria is their ability to multiply rapidly and operate more effectively in variable and less stable environmental conditions, unlike fungi, which typically require a more controlled environment for effective degradation of polymeric materials [34]. Some algae species also demonstrate the ability to degrade difficult-to-degrade polymers [60,66]. Algae can adhere to plastic surfaces, initiating the biodegradation process by secreting exopolysaccharides and ligninolytic enzymes. This ability is particularly important in environments where other microorganisms have limited adaptive capacity, such as in marine ecosystems, where a significant portion of plastic waste is concentrated [34]. However, unlike bacteria, algae use atmospheric carbon dioxide as their primary carbon source and sunlight as their primary energy source. Despite their ability to colonize plastic surfaces and assimilate MPs, their metabolic pathways do not support the full mineralization of these compounds. This fact is of concern because ineffective degradation promotes the bioaccumulation of MPs and their further penetration into the food chain [14].

The formation of biofilms on the surface of the tested polymers is considered one of the first stages of the biodegradation process [41]. Microorganisms modify and consume the polymer, using it as an energy source, which in turn changes its properties, leading to mass loss, structural changes, and ultimately, carbon bond formation [18]. Carbon-carbon bonds in the chains of many polymers give the plastics a compact structure, which translates into very slow degradation under natural conditions [3]. Generally speaking, microorganisms cannot degrade plastics composed solely of carbon and hydrogen atoms because they lack sites containing carbon-oxygen bonds (C=O, C-OR, C-OH), which are targets for enzymes produced by microbes [54]. The lack of degradation potential is an important feature for products intended for long and repeated use, but it is not desirable for the production of disposable materials. It has been estimated that the average use time of a plastic bag is only 12 min, despite its potential for repeated use. SUP products, once discarded, can remain in the environment for up to a thousand years. This creates a huge disparity between their useful life and their decomposition, leading to the world currently being drowned in plastic [3], with plastics now a defining component of the current geological era. Vast quantities of plastic waste have created a new microbiological habitat known as the plastisphere [4]. These communities support the formation of polymicrobial biofilms that contribute to degradation, but as separate entities, they can also pose an additional threat, for example, through the generation of drug-resistant bacteria (polymicrobial populations enable the acquisition of additional traits, such as resistance to antibiotics and other antimicrobial substances, especially in areas with high chemical concentrations) or the attachment of other human pathogens [67].

The rate of polymer degradation can be measured using various convenient analytical and comparative methods (Table 2). These methods enable the assessment of the material’s state before and after the decomposition process, the analysis and modification of functional groups, the examination of the formed biofilms, the analysis of the surface, or the visualization of changes indicating fragmentation or changes in the structure and nature of the material [41].

When considering the degradation of polymers from the food sector, they can be divided into the so-called non-degradable polymers, which have a C–C backbone (e.g., PE, PP, PS [37]), and the degradable ones, which contain hydrolyzable bonds in their backbone (e.g., PET) [40].

### 3.1. Polyethylene Degradation

#### 3.1.1. Degradation Mechanisms

Research on the degradation of plastics, particularly polyethylene, has been conducted for years. Physicochemical, microbiological methods, and combined methods are used. Degradation of this polymer is difficult and challenging. Storage in moist soil for 12–32 years revealed only slight degradation and a small weight loss [18]. Resistance to degradation is primarily due to its high molecular weight, absence of functional groups, degree of crystallinity, hydrophobicity, and insolubility in aqueous solutions. The initial cleavage of PE in the natural environment is due to the synergistic effects of abiotic and biotic factors. A mechanism for PE degradation has been proposed based on four stages: abiotic treatment/biodeterioration, biofragmentation/depolymerization, assimilation, and mineralization [40]. In the first stage, the plastic is exposed to UV light. The radiation is initially absorbed by the polymer chain, generating radicals. Ultimately, oxygen is absorbed, and hydroperoxides are formed, inducing the formation of carbonyl groups. Photooxidation can be initiated by contaminants and pro-oxidants present. UV can also initiate degradation at sites containing trace amounts of hydroperoxide or ketone groups introduced during the PE manufacturing or processing [75]. The material can then be subjected to heat treatment or chemical oxidation, the purpose of which is to generate unstable superoxide radicals, which makes it susceptible to further transformations by microbial enzymes [40]. Thermal treatment and UV light have been shown to reduce chain length and create surface oxidized carboxyl, hydroxyl, and carbonyl groups [18]. The formation of surface carbonyl groups is considered the initial stage of biodegradation. These modifications alter the crystallinity and structure of the polymer, facilitating its degradation. In addition to abiotic factors, initial oxidation can also be the result of microbial activity and is referred to as biodeterioration. Biotic and abiotic oxidation processes modify PE structure, leading to the formation of oxidized oligomers that are more easily biofragmented and depolymerized. This results in shorter oxidized segments with 10–50 carbon atoms that can be transported into microbial cells, where further assimilation occurs in metabolic pathways. When the process occurs under aerobic conditions, the polymers are ultimately converted to CO_2_ and biomass via metabolic pathways [40]. Over the years, various microorganisms capable of forming surface biofilms have been studied and are believed to be capable of biodegrading PE (Table 2). The initial stage of biodegradation involves the hydroxylation of carbon-carbon bonds, resulting in the formation of primary or secondary alcohols. These compounds are then oxidized to aldehydes or ketones, and then to carboxylic acids. As a result of this microbial oxidation, the number of carbonyl groups is reduced as they are converted to carboxyl groups. Carboxylated n-alkanes are structurally similar to fatty acids, which bacteria can degrade via the β-oxidation pathway. Knowledge of the genetic mechanisms of PE degradation remains very limited. Alkane hydroxylases (AlkB), enzymes involved in the alkane degradation pathway, play a key role in the degradation of linear alkanes and are the best-known enzymes involved in PE degradation via β-oxidation. Monooxygenases, the main enzymes of the alkane hydroxylase system, are particularly important in this pathway. The number and types of alkane hydroxylases vary depending on bacterial species, induction conditions, and target alkane chain length [75]. Enzymes involved in PE degradation include manganese peroxidase (MnP), which reduces tensile strength and average molecular weight; soybean peroxidase (SBP), which oxidizes the surface and increases hydrophobicity in combination with hydrogen peroxide; and laccase (Lac), which oxidizes the surface and reduces molecular weight [42]. When considering PE degradation, it is important to remember the significant impact of production additives. These additives, often degradable, contribute to material fragmentation and can interfere with the effective assessment of PE degradation itself. Various additives are intentionally incorporated into polymeric materials to accelerate degradation, such as compounds that render them oxo-degradable, and are then referred to as addition polymers. These additives include polyunsaturated compounds, transition metals (cobalt, iron, calcium, and manganese), natural polymers (cellulose, starch, chitosan), food dyes, or other synthetic polymers with hydroxyl, ester, or ether groups susceptible to microbial hydrolysis [75]. Despite numerous studies and identification of many strains with PE degradation potential, research remains largely observational, and more detailed analyses are needed to precisely determine the PE degradation pathways [40].

#### 3.1.2. Toxicological and Processing Aspects Related to PE Degradation

The widespread use of polyethylene materials poses serious ecological threats to both terrestrial and marine ecosystems. One example is the blockage of the digestive tracts of fish, birds, and marine mammals. Furthermore, hundreds of species from various ecosystems are at risk due to the consumption of this waste, leading to wide-ranging consequences for biodiversity [18]. Due to its low density, PE is mainly found at relatively shallow depths in the aquatic environment (3–100 m), corresponding to the habitat of many aquatic organisms. This poses a significant ecological threat [76]. On land, tons of unprocessed PE waste lead to, among other things, a reduction in soil quality and physiological disturbances in plant growth, such as reduced seed germination and inhibited root growth, thus also resulting in a decrease in agricultural productivity. Therefore, the processing of this type of waste is currently a global problem [18]. PE has become a major environmental pollutant, constituting 50% of total MP waste [69]. Mechanical recycling of PE is relatively well developed compared to most other plastics (except PET), but the process requires washing and sorting of the material. Closed-loop recycling, which would allow the reuse of the processed material for the same application, is rarely practiced for collected polymer waste. This is due to several key challenges: first, the loss of properties due to repeated processing; second, the complexity of the species, i.e., the existence of many different types of PE, which significantly complicates the mechanical recycling process. Furthermore, different PE products contain different stabilizers, processing aids, and other additives in varying amounts. Consequently, despite well-established mechanical recycling technology, achieving a true circular economy for PE remains a challenge [77]. Chemical recycling, which involves depolymerization to monomer, is a promising alternative to mechanical recycling because it preserves the high-quality properties of the recovered material. PE is characterized by linear hydrocarbon chains, allowing crystalline packing and excellent performance properties. Unfortunately, the chemical inertness of PE hinders its chemical recycling. This process requires extremely high temperatures, exceeding 600 °C, and ethylene recovery is low, less than 10%. These demanding conditions currently make chemical recycling of PE an economic and technological challenge [78].

### 3.2. Polypropylene Degradation

#### 3.2.1. Degradation Mechanisms

PP degradation is similar to PE [41]. Like PE, PP is a highly hydrophobic hydrocarbon polymer. It also lacks accessible functional groups that could undergo hydrolysis or be targeted by microorganisms, making its degradation in the natural environment extremely slow. Biodegradation of branched isoparaffins is more difficult than that of linear paraffins [25]. The presence of methyl side groups in each repeat unit, combined with the high molar mass, makes PP biodegrade more complexly than PE. The lower degree of biodegradation may result from poor penetration of PP particles through microbial cell membranes [79]. The primary mechanism of PP degradation is photooxidation induced by UV and visible light. This process leads to polymer chain scission, a decrease in tensile strength, and the separation of individual fibers, which become brittle and eventually disintegrate into micro- and nanoplastics. Photodegradation of PP occurs in stages: initiation, propagation, branching, and termination. During initiation, UV radiation is absorbed by PP molecules, generating free radicals. One of the most common radicals formed is the alkyl radical (–CH_2_–), which can react with other PP molecules, leading to chain scission. Another possible initiation product is the allyl radical (–CH_2_–CH=CH_2_), which, upon reaction with oxygen, forms hydroperoxides. During the propagation phase, free radicals react with neighboring PP molecules, generating additional radicals and leading to the formation of functional groups such as carbonyls and hydroperoxides. This process can occur in both the presence and absence of oxygen. PP can also undergo thermal and hydrolytic degradation. Thermal degradation breaks polymer chains into smaller fragments, resulting in a loss of mechanical properties. Hydrolytic degradation can be accelerated by factors such as moisture, elevated temperature, extreme pH, and catalysts, and may produce functional groups including carboxylic acids, hydroxyls, and ketones. However, both hydrolytic and thermal degradation of PP are extremely unlikely under typical environmental conditions, particularly in cold or marine environments, where accelerating factors are rare or limited [39]. Although it is known that PP can be degraded by various microorganisms, no key depolymerase enzyme directly responsible for its biodegradation has yet been identified. Therefore, a complete understanding of the mechanism of PP degradation from a microbiological perspective is lacking. It is assumed that the first step of microbial degradation involves hydroxylation of carbon-carbon (C–C) bonds in the long hydrocarbon chain, leading to the formation of primary or secondary alcohols. These compounds are then oxidized to aldehydes and ultimately to carboxylic acids. By converting the carbonyl groups to acid, oxidation reduces the number of carbonyl groups. Carboxylated alkanes are structurally similar to fatty acids and can be metabolized by bacteria via the β-oxidation pathway. This pathway produces acetyl coenzyme A (acetyl-CoA), which then enters the tricarboxylic acid cycle (TCA cycle), leading to the formation of fumarate. Studies also suggest that, similarly to polyethylene (PE), the use of biodegradable additives and physicochemical pre-treatment methods, such as UV radiation, gamma radiation, or thermo-oxidation, can significantly increase the susceptibility of PP to microbiological degradation [79].

#### 3.2.2. Toxicological and Processing Aspects Related to PP Degradation

Polypropylene waste can be incinerated, disposed of, or recycled. A very small fraction of PP reaching the market (less than 1%) is recycled [80]. This is mainly because PP is typically found in mixed waste streams, which makes its effective separation and processing difficult. A key barrier to effective recycling is the need for thorough washing and separation of the individual components of this waste. Where separation of the raw material is effective and feasible, mechanical recycling still presents significant limitations. Multiple melting and reprocessing cycles lead to rapid degradation of the PP molecular backbone. Thermal degradation is more severe than for PE because the PP backbone contains a tertiary carbon atom, which is particularly susceptible to thermo-oxidative and photo-oxidative degradation. Although some PP plastics contain stabilizers to prevent degradation, even after successful recycling, additional stabilizers must be added to ensure adequate oxidative stability of the newly produced material. However, the increasing presence of stabilizers and their degradation products can lead to a deterioration of the mechanical properties of PP. For example, the elongation at break for the original, unstabilized PP sample is 65%; after only 10 recycling cycles, this value drops to 45%. When PP becomes unsuitable for mechanical recycling (e.g., by grinding or melt processing), new strategies for converting it into higher-value-added raw materials are gaining importance, using technologies such as plasma reactors or supercritical water [22]. An alternative method for PP decomposition is waste pyrolysis. Pyrolysis is a thermochemical process in which plastic waste is converted into lower molecular weight compounds by decomposing it at high temperatures under oxygen-limited conditions. This method can produce high-quality products suitable for fuel applications. Recent studies have shown promising results regarding the increased yield of pyrolysis byproducts, including liquid oils, gases, and solid char products [81].

### 3.3. Polystyrene Degradation

#### 3.3.1. Degradation Mechanisms

Polystyrene is a material that is very difficult to degrade. Its lack of functional groups, hydrophobicity, high molecular weight, and macromolecular nature are the main factors inhibiting degradation [55]. The first biodegradation attempts date back to 1979, when a 0.5% degradation rate was demonstrated after 11 weeks of incubation with a mixture of microorganisms. Some studies showed no signs of degradation even after 32 years of burying a polystyrene sheet in soil. Improved biodegradability was achieved only by adding other biodegradable materials (starch, lignin, and mono- or disaccharides) to the raw material. Several microbial strains have also been isolated (Table 2) that demonstrated some degradation capacity towards PS [53]. Insects with promising properties for PS degradation have also been identified. For example, studies on *Tenebrio molitor* larvae showed that they could digest polystyrene and grow as well as the control group over a one-month test period. As a result of digestion, 47.7% of the consumed carbon from polystyrene was converted into CO_2_ [52]. *Achatina fulica* snails were also subjected to similar studies and demonstrated significant weight loss (30.7%), as well as their ability to depolymerize and biodegrade. However, the presence of numerous micro-PS particles in their feces was also noted, which is an unfavorable phenomenon considering their harmfulness [73]. Although the exact pathways and mechanisms of PS degradation are currently poorly understood and remain an area of research, the isolation of numerous organisms that can be used as potential degrading agents provides much optimism for solving the problem [40]. In recent years, a model of the PS degradation mechanism based on abiotic and biotic factors has been proposed. UV radiation damages the structure of PS and often manifests itself as yellowing. This photoyellowing is the result of reaction products formed during the process, which absorb radiation in the UV and short visible wavelengths [82]. The mechanism of PS photolysis in the solid state depends on the mobility of free radicals in the polymer matrix and their bimolecular recombination. Hydrogen free radicals are characterized by a high ability to diffuse within the matrix, allowing them to form radical pairs or abstract hydrogen atoms from polymer chains. Phenyl radicals, on the other hand, are much less mobile. However, they can interact with the surrounding environment, abstracting hydrogen atoms from nearby polymer fragments or reacting with polymer radicals or hydrogen radicals. Hydrolysis and subsequent oxidation also play a significant role [83]. Biodegradation can begin with cleavage of the main or side chain, leading to several degradation pathways. Decomposition of the polystyrene (PS) main chain can lead to the formation of various aromatic monomers, such as styrene, toluene, etc., which are then used as carbon sources in two proposed microbial catabolic pathways. In the first pathway, styrene is first converted to styrene oxide by styrene monooxygenase (SMO) and then metabolized to 4-maleylacetoacetate by the following enzymes: styrene oxide isomerase (SOI), phenylacetaldehyde dehydrogenase (PAALDH), phenylacetate hydroxylase (PAAH), 2-hydroxyphenylacetate hydroxylase (HPAAH), and homogentisate dioxygenase (HGADO). 4-maleylacetoacetate is further converted by the β-oxidation pathway to acetyl-CoA, which then enters the citric acid cycle (TCA), where it participates in central biosynthetic pathways. In the second proposed pathway, styrene is hydroxylated at the aromatic ring by styrene dioxygenase (SDO), leading to the formation of cis-styrene glycol. This compound is then converted to acetyl-CoA by the following enzymes: cis-glycol dehydrogenase (CGDH), catechol 2,3-dioxygenase (CDO), 2-hydroxymuconic semialdehyde hydrolase (HMASALDH), 2-hydroxypenta-2,4-dienoate hydratase (HPDEH), 4-hydroxy-2-oxovalerate aldolase (HOA), and pyruvate dehydrogenase complex (PDHC). The resulting acetyl-CoA also enters the TCA cycle, where it participates in the synthesis of biomass or the production or accumulation of other metabolites, such as poly(hydroxyalkanoic acid) (PHA) [40]. Other model studies suggest that oxidases or oxygenases may also participate in the cleavage of C–C bonds in the polystyrene (PS) structure, which contributes to the increased susceptibility of these polymers to biodegradation [51].

#### 3.3.2. Toxicological and Processing Aspects Related to PS Degradation

The mass production of PS, combined with inefficient waste management systems, has led to the rapid accumulation of polystyrene waste. Unfortunately, this waste, although recyclable, is often treated as garbage and ultimately ends up in landfills or is incinerated, contributing to serious ecological and environmental problems [26]. Combustion of polystyrene can release numerous pollutants into the atmosphere, including polycyclic aromatic hydrocarbons (PAHs), soot, carbon monoxide (CO), nitrogen oxides (NOx), and many other chemical byproducts [84]. The most common form of commercially available polystyrene is EPS, followed by foils. Expanded PS consists of approximately 95% air and only 5% polymer, which means its production is associated with significant air pollution and the generation of large amounts of solid and liquid waste. The EPS manufacturing process uses hydrochlorofluorocarbons (HCFC-22), which are greenhouse gases that damage the ozone layer. Once released into the atmosphere—at altitudes exceeding 16 km—HCFC-22 contributes to ozone depletion. PS used in food packaging poses a particular threat, as its production and use can release up to 3.9 tons of chlorofluorocarbons (CFCs) into the atmosphere. Furthermore, PS can indirectly contribute to ozone formation. When released to the ground, PS releases hydrocarbons, which, when reacting with nitrogen oxides (NOx), can lead to the formation of ground-level ozone. Although ozone in the upper atmosphere protects against UV radiation, its presence at ground level poses a serious health risk to humans, animals, and plants. Furthermore, according to the National Research Council’s 12th National Toxicology Program Carcinogens Report, styrene, the primary monomer used in the production of PS, has been classified as a probable human carcinogen. A possible link between exposure to it and the development of pancreatic, kidney, and esophageal cancers, as well as lymphomas and leukemias, has been identified. Although ingestion of PS particles in large quantities (up to 500 g/mL) does not demonstrate significant systemic toxicity, studies suggest that PS nanoparticles with a diameter below 1 μm may have negative biological effects, particularly on red blood cells. They have been shown to disrupt van der Waals forces between erythrocytes, leading to hemolysis, and also cause irritation to tissues and organs [25]. In response to the growing plastic pollution crisis, attempts have been made for many years to implement closed-loop mechanical recycling of PS. However, in practice, less than 5% of this material is recycled. This is due to the limitations of current mechanical recycling methods, which are expensive, prone to contamination, and lead to a reduction in the quality and value of the recovered materials [26]. In 2001, out of 25.4 million tons of plastic waste, 2.29 million tons of PS were produced, none of which was recycled, necessitating disposal. Over 34% of total PS production was disposable products, such as plastic cups and plates, approximately 26% went to everyday items, and 30% to long-life products. Because virgin PS is more durable and cheaper to produce than recycled material, reusing PS is becoming a significant yet difficult challenge. The cost of recycling one ton of PS is approximately $3000, which effectively discourages the market from investing in the recovery and reprocessing of this material [25]. Research on PS recycling focuses primarily on pyrolysis, chemical degradation, and the use of enzymes, which is a rapidly developing field of science. Pyrolysis, both thermal and catalytic, enables the depolymerization of polystyrene waste into styrene monomer. The resulting high-value raw material can be reused to produce pure PS or other functional materials. However, it should be noted that pyrolytic processes are highly energy-intensive and generate significant costs, which currently limit their widespread use on an industrial scale [26]. A key challenge in this area is to develop a scalable and energy-efficient process that will efficiently convert PS waste into a single, dominant end product. Such an approach would increase the cost-effectiveness of recycling and facilitate its implementation on an industrial scale [85].

### 3.4. Polyethylene Terephthalate Degradation

#### 3.4.1. Degradation Mechanisms

PET, as a polymer containing hydrolyzable bonds, should theoretically be more readily degradable. However, its structure makes it significantly more resistant to biological and chemical degradation. Although the ester bonds present in the PET structure are susceptible to hydrolysis, there are several reasons why the polymer exhibits greater resistance to degradation. Key factors include the degree of crystallinity, molecular weight, polymer chain flexibility, surface hydrophobicity, and hydrolysis reaction temperature. The stiffness of PET, resulting from the presence of aromatic terephthalate units in the material’s structure, is the main reason for its low biodegradability. Furthermore, the high content of these aromatic blocks limits the mobility of the polymer chain, significantly hindering its enzymatic degradation [30]. Under environmental conditions, PET is subject to numerous biotic and abiotic factors that contribute to its slow degradation. Photodegradation of PET occurs under the influence of light in the near-ultraviolet range, leading to the scission of the polymer chain via Norrish Type I and Type II reactions. Simultaneously, a cross-linking process occurs, resulting in the material becoming brittle, discolored, and characterized by an uneven surface. PET exposed to UV radiation degrades relatively rapidly, leading to a deterioration of its physical and mechanical properties and the development of an intense yellow color. The photooxidation mechanism of PET is proposed to involve the oxidation of methylene (CH_2_) groups adjacent to ester bonds, leading to the formation of hydroperoxide species, which then transform into various photoproducts. This process involves both the ester fragments of the terephthalate units and the methyl groups. The ends of the vinyl ester chains can act as cross-linking centers and gelation initiators. They polymerize and then undergo thermal degradation, forming conjugated polyene systems with a yellow or brown color, contributing to the material’s discoloration. This process is catalyzed by carboxyl groups, and an increase in the number of carboxyl ends correlates with an increased tendency of PET to discolor [32]. The degradation process is also highly temperature-dependent. The initial stage of thermal degradation involves the random cleavage of ester bonds within the polymer chain, leading to the formation of carboxyl end groups and vinyl ester structures. This is followed by a transesterification reaction of the vinyl ester, which produces vinyl alcohol, which is immediately converted to acetaldehyde. As a result, the polyester chain is regenerated, and the average degree of polymerization remains relatively unchanged. The net effect of this reaction is the conversion of the terminal hydroxyl groups to carboxyl groups, accompanied by the formation of acetaldehyde. Furthermore, degradation may be accompanied by radical processes; the abstraction of a hydrogen atom from the polymer chain, induced by the presence of impurities, leads to the formation of radicals. They react with oxygen to form peroxide radicals and then unstable hydroperoxides that are sensitive to heat and light, initiating subsequent stages of degradation [86]. Under conditions of elevated humidity, hydrolysis occurs. This process results in an increase in the number of terminal carboxyl and hydroxyl groups, as well as the formation of shorter polymer chain fragments, likely as a result of reverse esterification. The presence of an acidic or alkaline environment significantly accelerates the rate of hydrolysis, but the reaction mechanism itself is assumed to remain essentially the same. Water is assumed to diffuse primarily into the amorphous regions of the polymer, where the process occurs. The rate of the process depends on the morphology of the material, particularly its degree of crystallinity, but also on environmental conditions such as relative humidity and temperature. Furthermore, it has been shown that the kinetics of PET hydrolysis can be autocatalytic, meaning that the products formed during the process can further accelerate the reaction [87]. Microorganisms play a key role in the subsequent stages of degradation (Table 2). Literature data indicate that bacteria dominate among the microorganisms capable of PET degradation, accounting for 56.3% of cases. Fungi accounted for 32.4% of reports, while 7.0% of studies involved mixed systems (bacteria and fungi). Microalgae were used in 1.4% of cases, and the remaining 2.8% involved symbiotic microorganisms associated with invertebrates such as insects [59]. To depolymerize this material, microorganisms secrete extracellular enzymes that break down PET into water-soluble intermediates. These compounds are then used as substrates in metabolic processes, leading to further degradation. However, the presence of ester groups in the PET structure increases its resistance to biodegradation, which poses a significant challenge for effective biodegradation [64]. It is worth noting that enzymatic degradation is strongly dependent on the degree of PET crystallinity [33]. The discovery of the bacterium Ideonella sakaiensis and its ability to utilize PET as a sole carbon source has generated considerable interest in recent years. *I. sakaiensis* utilizes two enzymes—IsPETase and IsMHETase—to degrade PET into its basic monomers. To date, *I. sakaiensis* remains a unique example of an organism that performs extracellular PET degradation, enabling subsequent utilization of the degradation products in intracellular catabolic pathways [88]. The PETase enzyme, responsible for PET hydrolysis, catalyzes depolymerization, breaking down the polymer into mono(2-hydroxyethyl)terephthalic acid (MHET), with the simultaneous formation of trace amounts of terephthalic acid (TPA) and bis(2-hydroxyethyl)terephthalate (BHET) as byproducts. The MHETase enzyme then converts MHET into two basic monomers: terephthalic acid (TPA) and ethylene glycol (EG) [57]. The amounts of the resulting end products depend on the reaction carried out by PETase. Similar degradative activity has been found in cutinases from various members of the genus *Thermobifida* and other microbial species, but they were found to be less efficient [88]. It is assumed that enzymatic hydrolysis of PET occurs most effectively at temperatures close to the glass transition temperature (Tg) of PET, which is approximately 65–80 °C. Under these conditions, polymer chains in amorphous regions of PET acquire sufficient mobility, allowing enzymes (PET hydrolases) to access their active sites and carry out the degradation reaction [30]. Active enzymes used for PET depolymerization are characterized by low catalytic activity, which significantly limits their practical application in commercial biodegradation processes. Therefore, enzyme activity has been increased biotechnologically by introducing various genetic mutations that improve their stability, substrate specificity, and catalytic efficiency, enabling their use in recycling [89].

#### 3.4.2. Toxicological and Processing Aspects Related to PET Degradation

Although PET is widely considered chemically inert and safe for consumer applications, potential toxicological hazards are primarily related to the migration of antimony compounds (Sb_2_O_3_ catalyst), for example, into bottled water, especially after exposure to elevated temperatures [30]. Additionally, phthalates have been classified as toxic and potentially endocrine-disrupting substances. PET waste present in the aquatic environment is often consumed by fish and other marine organisms, which promotes its bioaccumulation and entry into the food chain. Furthermore, the presence of plastic fragments in agricultural soils can pose a threat to the quality of drinking water resources and contribute to the formation of MPs [90]. Despite PET’s recyclable properties, such as a high melting point, the ability to be processed without the need for additives, and the relative ease of separation, its primary application, i.e., beverage bottles, results in only about 30% of global PET production being recycled. PET recycling is carried out using various technologies, such as mechanical, chemical, enzymatic, and pyrolysis. The most commonly used method is mechanical recycling, which is characterized by low energy consumption, relatively low costs, and the ability to obtain materials of decent quality [29]. From the perspective of input costs and the value of final products, both physical and chemical recycling of PET waste are considered attractive solutions. Physical recycling methods enable rapid and mass processing of used PET products, but the recovered materials often have reduced mechanical and chemical properties. Importantly, physically processed PET and its oligomers typically do not meet food safety standards, precluding their use in food contact products, in accordance with current regulations (e.g., EU Regulation 2022/1616). In comparison, chemical recycling of PET enables the recovery of highly purified monomers, such as terephthalic acid, dimethyl terephthalate (DMT), and ethylene glycol (EG), which can be reused to produce virgin PET. Furthermore, chemical recycling processes can yield other valuable products, such as hydrogen, potassium dimethylsalt, nanocarbons, covalent organic frameworks (COFs), and metal–organic frameworks (MOFs) [28]. The environmentally friendly biological degradation of the synthetic PET polymer into its basic monomers, terephthalic acid (TPA) and ethylene glycol (EG), is highly desirable, especially in the context of their potential for reuse. Bioremediation is one of the most promising approaches to reducing environmental pollution with plastics on a global scale [60]. When considering PET recycling, its purity should always be taken into account. Many additives and production process residues in the plastic structure pose a significant obstacle. For example, catalysts used in the synthesis of high-molecular-weight PET have significant drawbacks: they are non-biodegradable, often toxic to human health and the environment, and, most importantly, difficult to remove from the final product. Consequently, because the catalyst remains within the polymer structure, side reactions can occur during secondary processing or mechanical recycling, leading to deterioration of the material’s properties. For this reason, mechanically processed PET is most often used for the production of secondary products [27]. It is worth noting that the material, despite the presence of benzene groups in its structure, is characterized by high flammability and limited charring capacity, posing a significant environmental risk [91].

## 4. Microplastics

A few decades ago, the problem of plastics in the environment was perceived primarily as an esthetic issue, resulting from the spread of waste from landfills to other environments, such as water bodies, under the influence of wind. The subsequent effects were not considered at that time. Currently, food packaging is the dominant source of small plastic particles worldwide. These particles can pose a risk to humans, primarily through ingestion. Common packaging MPs can enter the gastrointestinal tract after a long period of environmental degradation, ultimately contaminating food products from various environments, especially aquatic ones, through contamination during the production and storage process, or through direct migration from the packaging material [92]. Due to the action of various environmental factors, such as ultraviolet radiation, dispersed plastics on a macroscale undergo further fragmentation. Although photooxidation is the main fragmentation mechanism, weathering processes occurring during the dispersion of plastics in soil or water bodies also significantly contribute to the formation of these small particles [76]. As plastics degrade, their size decreases significantly. While this phenomenon makes the problem less visible, it also becomes more hazardous to the environment and health [93]. Plastics present in the environment are classified according to particle size into four main categories: macroplastics—those larger than 2.5 cm; mesoplastics—those between 2.5 cm and 5 mm; microplastics—those between 1 µm and 5 mm; and nanoplastics—those between 1 µm and 100 nm [30]. MPs are typically defined as particles of anthropogenic origin, manufactured or processed, and have been the most studied size category of plastic pollutants for several decades. However, the terminology related to size classification remains ambiguous and inconsistent. Currently, there is no standardized definition of the lower size limit below which the term “microplastic” is used. In the scientific literature, MPs are also often described as particles ranging from 1 to 1000 µm [94]. In 2004, the term MPs was formally introduced and defined as microscopic plastic fragments that are common and accumulate in the oceans, and their widespread occurrence in marine waters and sediments worldwide has been documented [95,96]. Recently, nanoplastics have attracted increasing attention. They include particles formed by the further fragmentation of MPs as well as primary particles released directly due to human activity, particularly from the growing use of nanoplastics in everyday products. Although no universal, standardized definition of nanoplastics has yet been developed, they are typically defined as particles smaller than 1 µm. This approach stems primarily from the fact that at this scale, the material exhibits distinct physicochemical properties, typical of colloidal particles [94]. The environmental impact of nanoplastics may therefore differ significantly from that of MPs, also due to their much smaller size, which allows them to penetrate biological barriers and accumulate in the tissues and organs of living organisms [97]. Small plastic particles occur in various sizes and shapes, such as microspheres, pellets, fragments, fibers, filaments, granules, films, and microfoams [98,99]. Based on their source, MPs are divided into primary and secondary MPs. Primary MPs are intentionally produced by the industry as microspheres of various sizes, mainly for cosmetic and skincare applications, as exfoliants, and abrasives. These types of granules enter the environment primarily during the production process, but also during transport and use. Secondary MPs are formed by fragmentation of larger items due to environmental degradation of waste or as a result of human use [100].

### 4.1. Sources and Pathways of Dispersion

It has been proven that MPs constitute anthropogenic pollution of all ecosystems. The sources of MPs are mainly the household, industrial, and agricultural sectors. Landfills are places for depositing waste originating from all these sectors, which receive significant amounts of both primary and secondary MPs. The presence of other pollutants at landfills additionally promotes the adsorption of these substances onto the surface of these small particles. MPs can pass into leachate with rainwater, and in the case of improperly designed sealing systems, they can penetrate into groundwater or neighboring land areas. Landfills have also been identified as a potential source of MP emissions into the marine environment. The main sources of MPs in landfill leachate are solid waste and residues from wastewater treatment plants, such as sludge, fats, oils, and greases. Plastics deposited in landfills are subjected to mechanical processes, which lead to the formation of additional MPs. All waste in landfills undergoes a series of processing stages: initial aerobic biodegradation, transition from aerobic to anaerobic conditions, acid formation and hydrolysis phase, followed by methanogenesis, and finally maturation and stabilization. Each of these stages accelerates plastic degradation and promotes the formation of additional secondary MPs [93]. Soil contamination by MPs also occurs as a result of agricultural practices, such as the use of polyethylene plastic mulches, plastic tunnels, containers, packaging materials, nets, or wrapping films for silage bales, as well as the use of sewage sludge and irrigation with wastewater. These materials gradually degrade over time, breaking down into smaller fragments, and this process is accelerated by exposure to sunlight [99]. MPs present in soil modify its physicochemical properties, leading, among other things, to a reduction in water retention capacity and disruption of the nutrient cycling process [101]. The transport sector generates hazardous microparticles as a result of the wear of car tires, airplane tires, brake wear, and road markings. It is estimated that the emission of MPs originating from tire wear during driving is, on average, about 0.81 kg per person per year. Tire wear accounts for 5–10% of the global amount of plastic entering the marine environment. Additionally, particles generated by tire wear can constitute 3–7% of the suspended particulate matter fraction in the air [102]. The atmosphere plays a key role as a transport pathway for suspended particles, including MPs, to the surfaces of water bodies, urban areas, rural areas, and other remote locations. This transport occurs as a result of phenomena such as wind speed and direction, updrafts and downdrafts, convective lifting, and turbulence. It has been shown that, on average, 365 MP particles per square meter may settle from the atmosphere each day [103]. MP particles and synthetic fibers generated by human activities also enter wastewater treatment plants with domestic sewage [94]. It has been found that the inflow to wastewater treatment plants contains significant amounts of MPs, dominated mainly by microfibers from clothing washing and microbeads present in cosmetics and personal care products [103,104]. Due to their small size, conventional wastewater treatment processes have limited efficiency in capturing and completely removing them. Most chemical, biological, and physical methods studied for the removal of micro- and nanoplastics from water, such as dynamic membranes, membrane bioreactors, conventional coagulation, electrocoagulation, or biodegradation, encounter significant limitations that affect their practical effectiveness. A key problem is that many of these methods are non-destructive, which means that instead of breaking down micro- and nanoplastics, they merely transfer them to adsorbents or retain them on filters. Additionally, processes such as coagulation, flocculation, or electrocoagulation lead to the formation of large amounts of sludge containing high concentrations of plastics, creating further challenges for their disposal [94]. A significant portion of MPs originating from land areas enters aquatic ecosystems mainly through wastewater discharged from treatment plants and industrial effluents [103]. As a result, MPs reach rivers or settle in their sediments, and then enter the marine environment, where they can persist for decades. Transported by ocean currents, MPs spread on a large scale and are present not only in surface waters but also in sediments and the deep parts of the oceans [105]. It is estimated that 80% of MP pollution in the ocean originates on land, and rivers are the main channel through which plastic reaches the sea [106]. The discovery of the Great Pacific Garbage Patch (GPGP) vividly illustrates the scale of long-term pollution and devastation of the marine environment. It is estimated that annually, 1.15 to 2.41 million tons of plastic enter the oceans from rivers. These rivers contribute to the formation of five oceanic garbage patches, the largest being the GPGP, located in the Pacific Ocean. The GPGP currently covers an area of about 1.6 million square kilometers, which corresponds to an area three times larger than France. Plastic in the ocean undergoes very slow degradation, initially forming a gelatinous mass, and ultimately breaking down into MPs. In the central part of the GPGP, plastic concentration reaches hundreds of kg/km^2^, decreasing to about 10 kg/km^2^ in its most remote regions [107].

### 4.2. Conditions and Rate of Degradation

Fragmentation of macroplastics into microplastics constitutes the main cause of the presence of MPs in the environment. Despite the growing interest in this issue, knowledge about degradation mechanisms, changes in chemical composition, morphology, and the residence time of MPs in the environment remains limited. Due to their small size, MPs pose a significant ecological risk, as they can easily penetrate terrestrial and aquatic food chains through trophic networks. It is believed that this risk increases as particle size decreases, leading to an increase in the surface-to-volume ratio and thus to greater surface reactivity of MPs. Most MPs are formed as a result of abrasion of plastics during use, aging under atmospheric conditions, and the breakdown of larger plastic objects, leading to the formation of so-called secondary MPs. Although the basic processes leading to the fragmentation of plastics are known, detailed knowledge of the physicochemical stages of degradation occurring under real environmental conditions is still lacking. MPs, just like their larger counterparts, undergo very similar degradation mechanisms described above, with differences on the order of magnitude. MPs degrade much more slowly in natural environments, where mechanical impacts are less frequent and less forceful. An example is terrestrial areas, where mechanical stresses are mainly caused by wind. Particularly in more sheltered locations, this favors the maintenance of an intact photooxidative zone for a longer time. In the extreme case of no mechanical forces, degradation occurs homogeneously. A thin surface layer gradually decomposes into increasingly smaller and more polar macromolecules until their complete dissolution or mineralization to carbon dioxide. This process proceeds cyclically: after degradation of one subsurface layer, the next layer undergoes photooxidation. It is estimated that approximately every two years, a layer of 30 μm thickness is degraded, which translates into a total lifetime of MPs reaching even several decades in terrestrial conditions. The situation is completely different in dynamic environments, such as beaches, shorelines, rivers, or oceans, where MPs are subjected to a much greater number of collisions and shear stresses associated with turbulent flows. In such conditions, their lifetime is significantly shortened. After the induction phase, mechanical cracking of MP objects occurs, leading to continuous formation of new surfaces exposed to further degradation. It is suggested that the cracking process occurs cyclically, with a frequency of every six months, resulting in an exponential acceleration of MP degradation. In dynamic environments, the cracking of particles begins at an early stage of decomposition, leading to the formation of an increasing number of fine particles with sizes on the order of tens of micrometers. It is estimated that from a single MP particle, an average of about 500 smaller secondary particles may be formed during an average lifetime of 1.5 years in the environment. These particles, partially oxidized as a result of degradation, become hydrophilic, which favors the formation of biofilms on their surface by bacteria and other microorganisms and facilitates their uptake by aquatic organisms. Consequently, MP degradation products enter the food chain earlier, interacting almost immediately with flora and fauna [70]. Knowledge about the impact of biofilms formed on the surface of MPs on their degradation and removal from the ocean surface is still limited. In the marine environment, plastics with a density greater than seawater, such as PVC or PET, usually sink to the bottom. However, the fate of plastics with a density close to the surface seawater density, such as polystyrene (PS, 960–1040 kg/m^3^), is more difficult to predict due to the possibility of their suspension in the water column. Although biodegradation of plastic by bacteria colonizing MPs is documented, biofilms may simultaneously slow down this process by protecting the plastic from UV radiation or increasing the rate of particle sedimentation. Studies have shown that biofilm formation significantly slows down degradation processes, including by limiting the loss of phthalates (an indicator of PET degradation). Furthermore, it has been observed that the presence of biofilm increases the sinking velocity of both PET and PS. Research indicates that biofilm formation significantly affects both the rate of degradation and the transport properties of MPs in the marine environment [108]. Freshwater environments are also rich in plastic-degrading microorganisms, but their abundance is low. Studies on the behavior of MPs have shown that among freshwater ecosystems, eutrophic river sediments are characterized by the greatest degradation potential [95].

### 4.3. MPs as a Threat to the Environment and Living Organisms

**Figure 3:**
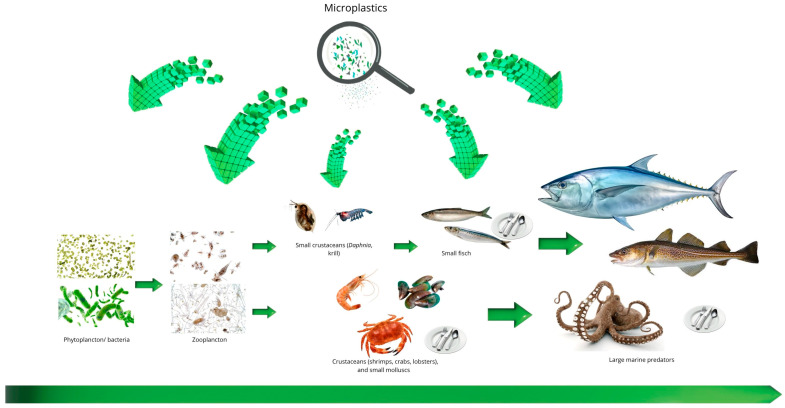
Trophic network illustrating the transfer of MPs in the marine ecosystem.

The continuous degradation of primary and secondary MPs leads to changes in their physicochemical properties, such as color, surface morphology, size, crystallinity, and density. These changes may affect their chemical reactivity, interactions with the environment, and potential effects on living organisms [98]. Due to the high degree of oxidation and the presence of peroxide groups, there is a high likelihood of adverse effects of these particles on living organisms. Since primary MPs act as a reservoir capable of continuously releasing hazardous fragments, a constant level of potentially toxic particles persists for many years. This duration depends on the intensity of solar radiation and the frequency of exposure to mechanical stresses [70]. MPs have a high capacity to adsorb toxic substances from the surrounding environment, which results from their small size and large surface-to-volume ratio. These substances include, among others, PAHs, polychlorinated biphenyls, DDT, HOCs, chlorinated pesticides, heavy metals (cadmium, lead, nickel, zinc), and compounds used during plastic production [109]. The sorption capacity of MPs depends on a number of factors, such as the presence of rubbery domains, particle size, specific surface area, functional groups, age, and polarity of MP particles, as well as the type of pollutants, including the polarity of organic pollutants, the presence of heavy metals, and the degree of hydrophobicity. Environmental parameters such as salinity, pH, and dissolved organic matter content also have a significant influence on the sorption process. Older MPs show a higher capacity to adsorb toxins compared to fresh or less degraded MPs [98]. Therefore, internalization of MPs may lead not only to direct physical damage in organisms but also to exposure to harmful chemical substances as a result of ingestion of contaminated MPs [14,97]. In addition, biofilms developing on plastic microparticles may be a source of harmful microorganisms and lead to the development of diseases in humans and animals [110]. At lower trophic levels, MPs may be mistaken for food by organisms due to their small size and color, leading to accidental ingestion. This increases the bioavailability of MPs in the food web. For example, MPs can be transferred from algae to zooplankton, and then to fish and seafood (Figure 3).

The color of MPs, most often yellow, white, or brown, resembles the natural food of zooplankton, making them easily recognizable and consumed. Consequently, MPs, through the food chain, can accumulate in aquatic organisms intended for human consumption [98]. Seafood accounts for 20% of animal protein for humans worldwide, and fish provide 15% of their intake. In the case of fish, the presence of MPs in the gastrointestinal tract does not suggest exposure for humans, since this organ is not usually consumed. However, MPs also accumulate in edible tissue as a result of transport, e.g., from the gastrointestinal tract. In addition to muscle tissue, the skin and gill tissue also pose a risk [109]. The ingestion of MPs by marine organisms leads to a range of negative effects, including mechanical damage to the gastrointestinal tract, blockages, and injuries to internal organs. The presence of MPs in the gastrointestinal tract of animals also causes malnutrition, as it fills the stomach without providing any nutrients. Moreover, MPs may disrupt the functioning of the reproductive system, resulting in reduced reproductive rates and decreased population sizes. Studies also show that MPs negatively affect the behavior and physiology of marine organisms, among others, by reducing their locomotor abilities and increasing their susceptibility to predators. Additionally, the accumulation of MPs in fish and crustacean tissues reduces their quality and commercial value, has raised concerns regarding food safety among consumers, and may negatively impact the economic viability of the fisheries sector [98].

In addition to waterborne MPs, which enter our diet through the consumption of fish and seafood and may be contaminated by leaching pollutants and additives present in MPs, direct human exposure to MPs from other sources, such as tap or bottled water and packaged food, should also be considered (Table 3) [111]. 

Food storage can be a significant source of MPs in the human diet. An increasingly urbanized lifestyle and the associated changes in consumption patterns may therefore result in increased exposure to MPs in processed foods in developed regions worldwide [129]. Common packaging materials such as PET, PS, PP, and PE are susceptible to mechanical abrasion, temperature fluctuations, and chemical leaching [130]. A concerning phenomenon in this regard is migration. Monomers, as components of packaging polymers, can migrate from the packaging material into the food product. This process often affects the aroma, odor, and taste of the product, ultimately leading to accumulation in the human body [92]. Food contamination by MPs can also occur through contact with plastic containers for beverages and food, such as bowls, trays, bags, and cups. Surface flaking of containers releases MPs, also as a result of various processing methods. It has been demonstrated that among takeaway containers, PS boxes release the most MPs during so-called “hot” processing. Polyethylene (HDPE) bags exposed to high temperatures also release MPs at concentrations twice as high as during cold processing. This suggests a correlation between increased MP release and rising temperature [129]. This relationship is further confirmed by a study showing much higher release levels (100-fold) of PP MPs from baby bottles filled with hot water compared to room-temperature water [131]. MP generation resulting from varying storage and washing conditions illustrates typical consumer usage. Abrasive processes occurring during food preparation and storage are particularly important in this context [129]. The presence of MPs in food is also influenced by the liners in cans and vacuum packaging, whose quality deteriorates over time [130]. During the use of food in plastic packaging, factors such as temperature and UV light have a significant impact on the chemical stability of the packaging. In addition, liquid products can interact with the interior surface of the packaging. Factors such as acidity, viscosity, water content, fat content, and other properties dependent on the nature of the packaged product are particularly relevant. All these factors affect the quantity and rate of MP release [132]. Studies indicate that PS is the most commonly leached polymer from packaging materials, likely due to its production process, which results in a relatively loose structure and rough surface that accelerates particle release. PP and PET are produced with smooth surfaces but are also susceptible to degradation. It should be noted that MPs are released even after simple rinsing of the interior parts of packaging, suggesting that even minor mechanical forces can contribute to increasing the MP load in food [133].

## 5. Effects of MPs on the Gastrointestinal Tract

**Figure 4:**
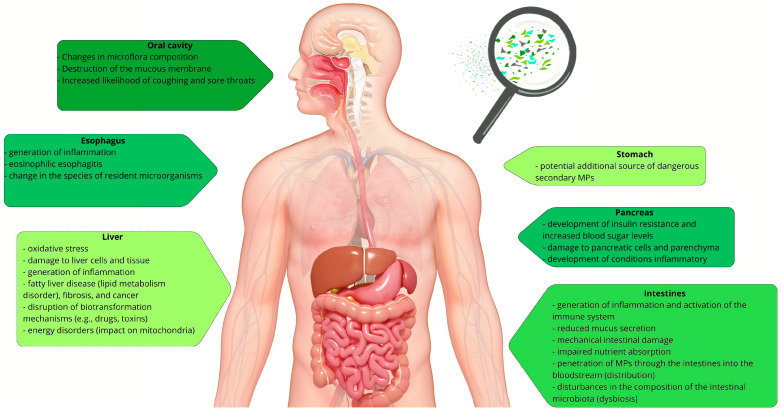
Effects of MPs on different components of the gastrointestinal system.

The gastrointestinal tract is one of the eight major systems of the human body, performing essential physiological functions such as the intake, transport, and digestion of food, absorption of nutrients, and excretion of waste products. Diseases of this system are characterized by high incidence, a strong tendency to recur, and significant mortality, resulting in substantial economic burdens for both society and individuals. With societal development, an increasing number of novel contaminants have gradually entered the human body through the gastrointestinal tract. This can lead to the development of various disorders, including gastritis, peptic ulcers, inflammatory bowel diseases, non-alcoholic fatty liver disease, pancreatitis, and metabolic disorders. Diseases of the digestive system have diverse causes and mechanisms, and MPs may contribute to their occurrence to some extent (Figure 4). MPs are transported and absorbed in the gastrointestinal tract through the Peyer’s patches of the ileum, and their harmful effects on the human body can be categorized into three main areas: inducing apoptosis of normal human cells or regulating gene expression via various signaling pathways; acting as carriers of toxic chemical substances; and functioning as foreign bodies that stimulate the organism and trigger oxidative stress responses, leading to the destruction of normal cells [134]. MPs can enter the human body through various routes: ingestion, inhalation, and skin contact. Among these routes, ingestion is the most common form of exposure. MP particles, often carrying contaminants, can be present in contaminated products, especially of marine origin, but also terrestrial foods, drinking water, or plastic food packaging and utensils [135].

Many studies report that the most contaminated product by MPs is mussels. This is certainly contributed to by the fact that these organisms act as water filters, accumulating significant amounts of hazardous compounds from the water. It is assumed that over the course of a year, dietary exposure may amount to approximately 11,000 particles per person as a result of consuming mussels and oysters by Europeans. Of course, the source of the consumed seafood, which correlates with the level of contamination, is significant in this matter [136]. After ingestion, MPs can potentially accumulate in the gastrointestinal tract and also translocate to other organs [101]. Studies conducted on human cell lines, rodents, and aquatic organisms indicate that MPs smaller than 10 µm can move from the intestinal lumen to the lymphatic and circulatory systems, leading to systemic exposure and accumulation in tissues such as the liver, kidneys, or brain. The smallest particles (<0.1 µm) can cross cell membranes, the placental barrier, and the blood–brain barrier, potentially allowing them access to all organs. Nevertheless, complete data regarding the absorption, distribution, metabolism, and excretion of these particles are still lacking. It is also unclear whether MPs cause dose-dependent effects in humans [110]. According to estimates, annual human exposure to MPs through food and beverage consumption may reach approximately 52,000 particles [96]. Focusing on MPs entering the body via the gastrointestinal route, air pollution cannot be overlooked. Plastic particles have been shown to be a significant component of fine particulate matter in the air, including indoor environments where food is present. Particular concern arises from the direct ingestion of household dust and dust deposited on food. It is estimated that household dust intake during meals could contribute a maximum annual exposure of 13,731–68,415 MP particles per person [136]. Larger particles are likely excreted via feces or, if deposited in the respiratory tract or lungs, removed through mucociliary clearance into the gastrointestinal tract. Limited data suggest that only a small fraction of ingested MPs is capable of crossing the epithelial barriers of the lungs and intestines. This process exhibits specific uptake profiles, with the efficiency of translocation increasing as particle size decreases [110]. Current research on the smallest particles clearly indicates that humans consume, and will continue to consume, plastics on an ongoing basis. The greatest concern is regular exposure through the penetration of MPs into the intestinal epithelium. Ingestion of MPs poses a challenge to the gastrointestinal lumen, related to the rate and capacity of absorption, and often also to the transformation of the ingested microplastics. Individual MPs can interact with molecules of the digestive system at every segment. MPs may interact with compounds such as proteins, carbohydrates, lipids, nucleic acids, ions, and water, causing various changes and responses in the body [136].

The first point of contact between MPs and the gastrointestinal tract is the oral cavity. MPs may influence the composition of the oral microbiota and cause damage to its mucosal lining. The oral microbiome is the second largest microbial community in the human body, and its composition is crucial for many aspects of health. Commensal microorganisms maintain proper acid–base balance and prevent the development of inflammatory conditions. Increased exposure to MPs has been observed to raise the likelihood of cough and pharyngeal inflammation, as well as induce oxidative stress, potentially leading to chronic inflammatory states. The generation of inflammatory mediators correlates with damage to the oral mucosa, increasing its permeability to contaminants, including MPs [137].

The next stage of MPs along the gastrointestinal tract is the esophagus. Although no studies have yet examined the presence of MPs in humans, animal data suggest that MPs may induce inflammation, eosinophilic esophagitis, or shifts in the resident esophageal microbiota [134]. The transit time of MPs in the esophagus is relatively short, which likely results in a lower particle load compared to the stomach. The reduced abundance of MPs may also be influenced by peristaltic activity, gravitational forces, and the smaller surface area relative to other sections of the digestive tract [138].

From the esophagus, MPs pass into the stomach. The stomach is characterized by an exceptionally low pH (1.5–3.5) due to the presence of hydrochloric acid. While degradation of various plastic fiber types has been demonstrated under conditions resembling those in the stomach, direct evidence of such degradation in vivo is lacking. The potential additional breakdown into smaller particles raises further concerns, as it may increase the risk of absorption and accumulation, creating yet another source of MPs. Detection of MP fragments in the highly acidic environment of human stomachs post-mortem does not conclusively indicate their degradation or transformation, but it also cannot exclude it. These findings underscore the urgent need for further research [139].

MPs then move into the intestines. Numerous studies have confirmed the presence and accumulation of MPs in the intestines of various species. MPs larger than 150 µm are not absorbed; however, they adhere to the intestinal mucus layer and interact with the apical surfaces of enterocytes, potentially triggering inflammation by activating the immune system. Smaller particles (<150 µm) can cross the intestinal mucus barrier through several mechanisms: endocytosis by intestinal epithelial cells, transcytosis via microfold (M) cells in lymphoid tissue, persorption (transport through gaps formed after enterocyte loss in the distal portion of villi), and extracellular uptake. The primary sites of MP adsorption are the Peyer’s patches. Although intestinal absorption of MPs is considered relatively inefficient, it may still lead to systemic and chronic toxicological exposure [140]. For example, PS particles ≤20 µm exhibit intestinal absorption rates of 0.1–1% [141]. Oxidative damage to the intestines is currently considered a key mechanism of MP toxicity [140]. Studies on mammals have shown that PS MPs reduce mucus secretion and decrease the transcript levels of genes associated with mucin expression in the colon [142]. Other studies have demonstrated the effects of MPs on crypt proliferation and differentiation, accelerated development of colitis with severe body weight loss, diarrhea, and blood in the stool. MPs also caused macroscopic pathological intestinal damage and induced inflammation, disrupting the balance between epithelial self-renewal and differentiation in the colon [143]. MPs can occupy the intestinal lumen, interfering with the normal physiological absorption of nutrients and disrupting the function of symbiotic microbiota. Fine synthetic particles abrade the gastrointestinal tract, causing mechanical damage and generating additional inflammation. MPs induce apoptosis of intestinal epithelial cells through oxidative stress, increasing intestinal permeability. Moreover, they serve as carriers of hazardous substances and pathogens, which further affect the intestines and their resident microorganisms [134]. Many animal studies have examined alterations in gut microbiota composition due to MP exposure. While individual studies did not reveal consistent directional changes—likely due to differences in species, MP type, particle size, and exposure levels—all reports indicate dysbiosis. Changes in microbial diversity and composition impair proper physiological function [140]. Data on the impact of MPs on human gut microbiota are limited. In vitro studies by Tamargo et al. demonstrated that MP-PET particles or their potential monomers negatively affect gut microbiota, reducing the total number of aerobic and anaerobic bacteria, as well as *Bifidobacterium* spp. and *Clostridium*. 16S rRNA sequencing provided additional insights into biodiversity trends across taxonomic levels, revealing lower levels of *Bacteroides*, *Parabacteroides*, and *Alistipes*, alongside an increased proportion of *Firmicutes*. These findings suggest that MP-PET may exert significant antibacterial effects, disrupting the essential balance of gut microbial communities and affecting immunological homeostasis and barrier function. Such alterations are associated with systemic and intestinal diseases, including inflammatory bowel disease and irritable bowel syndrome. Changes in the *Firmicutes*/*Bacteroidetes* ratio are important indicators of human physiology and are commonly used as biomarkers for metabolic disorders such as obesity and diabetes. Increases in *Proteobacteria*/*Desulfobacterota*, *Escherichia*/*Shigella*, and *Bilophila*, along with decreases in other Enterobacteria, have also been observed. However, these studies considered only the effects of MP-PET without additional additives or contaminants, which may have further impacts on microbiota. Furthermore, actual human exposure involves an undefined mixture of MPs, making comprehensive studies challenging. Nonetheless, demonstrated disruptions in normal gut microbiota are critical for proper human function, and imbalances among microbial members underlie many diseases. The presence of MPs in the gut, resulting in clear dysbiosis, is therefore a key factor in the development of multiple pathologies [144].

Numerous studies report that the liver may serve as a potential target organ for MPs. The liver performs critical metabolic and storage functions and is responsible for detoxification, deoxygenation, lipid metabolism, and protein synthesis [145]. Animal studies suggest that MPs can cross the intestinal barrier and accumulate in the liver via the circulatory system. MP toxicity is reported to increase with longer exposure duration, smaller particle size, polymer type, and earlier life stages, with PS MPs representing the greatest threat. The harmful effects of PS MPs on the liver are primarily manifested through oxidative stress, characterized by decreased activity of superoxide dismutase (SOD) and catalase, accompanied by increased levels of malondialdehyde (MDA) [146]. Oxidative stress underlies MP-induced hepatic toxicity. MPs penetrate lipid layers via surface receptors, altering membrane structure, and are subsequently internalized through endocytosis. Once internalized, MPs interact with hepatocytes, leading to elevated production of reactive oxygen species (ROS). Excess ROSs contribute to lipid peroxidation and oxidative damage, impairing hepatic cells and tissue. Intercellular toxicity from MP exposure is further reflected by elevated liver enzyme levels (ALT, AST, LDH), released from damaged hepatocytes during liver injury [145]. MPs also contribute to the generation of inflammation (increased IL-1β and TNF-α), hepatic steatosis (with upregulation of cytochrome P4502E1 as a major catalyst for lipid peroxidation), lipid metabolism disorders (enhanced lipid accumulation, serving as carriers for fatty acid transport during endocytosis, promoting steatosis), and impaired biotransformation mechanisms (MPs can affect CYP450 enzymes involved in the metabolism of most drugs and toxins). Additionally, MPs can accumulate in mitochondria, inducing mitophagy via kinase signaling pathways, inhibiting mitochondrial function, and impairing energy supply, thereby reducing hepatic metabolic activity. Collectively, these effects contribute to overall hepatotoxicity [146,147].

The pancreas, as the primary organ regulating blood glucose levels, is also susceptible to the accumulation of plastic particles. Animal studies indicate that exposure to MPs leads to increased blood glucose levels. Prolonged administration of small doses of PS nanoparticles (PS-NPs) in mice results in their accumulation, induces oxidative stress, and promotes excessive ROS production, which in turn activates PI3K/Akt signaling pathways, contributing to the development of insulin resistance and consequently elevated blood glucose levels. The presence of MPs in pancreatic tissue also causes direct oxidative damage to pancreatic cells [148]. Studies conducted in pigs have shown that MP-PET can affect physiological processes in the pancreas at both low and high concentrations. MP-PET exposure increased blood insulin levels, risk of insulin resistance, and pancreatic inflammation [149]. Studies in fish exposed to MPs demonstrated that pancreatic tissue undergoes modifications depending on exposure. Observed changes included acinar morphology deformation, acinar contraction, increased inflammatory cell infiltration, substantial lipid accumulation, and, in severe cases, necrotic changes, hemorrhage, and capillary obstruction [150]. These findings suggest that the presence of MPs in human pancreatic tissue may induce similar effects depending on the type, size, and exposure duration of MPs.

Currently, there is a lack of studies on the impact of MPs throughout their entire passage through the gastrointestinal tract. It should be assumed that particles entering the body undergo further fragmentation and biotransformation, although only a few studies have addressed this. One example is a study investigating the effects of MP-PET using a gastrointestinal model designed to replicate colon microbiota responses. The study examined MP exposure at concentrations close to realistic environmental intake (166 mg), corresponding to estimated daily PET consumption, and focused on potential changes occurring during the passage of MPs through the entire gastrointestinal tract. The transit of MP-PET through the gastrointestinal tract resulted in relative amorphization of the polymer structure without significant morphological changes. Deposits of salts (from gastric and intestinal fluids) and organic matter (protein corona, including enzymes) were observed on the particle surface. After passage through the colon, PET surfaces contained organic deposits and microbial biofilms from the resident microbiota. The crystalline structure of MP-PET changed during colon passage, confirming polymer biotransformation as a result of digestive processes, and progressive amorphization suggests biodegradation likely driven by gut microbiota. Such studies indicate that other MPs may also undergo structural changes during gastrointestinal transit, depending on multiple factors [144].

## 6. Conclusions

Since the 1950s, approximately 8.3 billion tons of plastic have been produced globally. Only 9% of this amount has been recycled, and 12% has been incinerated, while the remaining 79% has accumulated in the environment, revealing serious shortcomings in traditional waste management systems that fail to ensure sustainable plastic management. If current trends continue, it is predicted that by 2050, approximately 12 billion tons of plastic waste will end up in landfills or natural ecosystems, with a significant portion coming from food packaging [107]. The main sources of packaging materials are non-renewable resources such as oil and natural gas, the extraction and processing of which cause significant environmental degradation, greenhouse gas emissions, and health risks [3]. Although plastic packaging offers significant benefits for food preservation and distribution, excessive single-use packaging generates waste that is difficult and expensive to recycle [107]. Enzymes capable of degrading both biodegradable and durable plastics offer a promising alternative, and current research is focused on understanding and optimizing enzymatic degradation pathways through bacterial and fungal enzymes [151]. To reduce plastic waste, strategies must prioritize waste reduction at the source, including innovative retail practices that eliminate unnecessary packaging and encourage responsible consumption. These waste avoidance strategies are fundamental for reducing the negative environmental impact of packaging [107]. A variety of biopolymers are currently being developed to serve as substitutes for traditional fossil-based plastics. This is driven not only by the depletion of fossil resources but also by growing waste piles, environmental pollution, and the need to transform vast amounts of waste from various origins and compositions into value-added solutions [5]. There are three alternatives to conventional plastic applications: biodegradable plastics, compostable plastics, and bioplastics [4]. Biopolymers such as polylactic acid (PLA), polyhydroxyalkanoates (PHA), and cellulose films offer significant potential. These materials, while promising, face limitations such as high production costs, limited degradability, and recycling challenges [4]. Advanced packaging solutions using non-polymeric coatings or nanotechnology barriers can also help reduce MPs migration and ensure food safety. Achieving sustainable implementation requires collaboration between scientists, policymakers, and industry [130]. Recent EU legislation introducing a €0.80 tax per kilogram of non-recycled plastic aims to promote a circular economy and increase recycling efficiency [152]. The extensive use of plastics in the food sector contributes to the growing problem of MP pollution. Through photochemical, chemical, physical, and biological degradation, plastics break down into MP particles, which are ubiquitous in the environment, food, and human tissue [153]. Their potential toxicity is related not only to their small particle size and high surface reactivity but also to their role as carriers of environmental contaminants and additives such as plasticizers and flame retardants [154]. Ingested MPs interact with the gastrointestinal system at multiple stages—from the mouth to the intestines—where they can disrupt the gut microbiota, increase intestinal permeability, and contribute to inflammation, ulceration, and potentially carcinogenesis [155]. Evidence suggests their association with gastrointestinal, genotoxic, and metabolic disorders, including obesity, diabetes, allergies, and cancer, which are common problems affecting people worldwide [11].

## 7. Perspectives

Despite the continuously increasing number of scientific publications concerning the problem of MPs, many issues still remain unexplained. Almost all aspects remain subjects for long-term research. Current literature does not clearly emphasize that MPs are harmful, nor do they confirm their safety. However, all available studies suggest the great significance of this problem in a global context [156]. Current studies on the effects of MPs on the gastrointestinal tract remain limited to high concentrations, single species, specific particle sizes, and acute exposure conditions. The absorption and accumulation of MPs in non-laboratory environments represent a complex, long-term, and chronic process. The complexity of diverse environmental conditions also lies in the fact that contaminants often coexist with other substances (e.g., heavy metals, polycyclic aromatic hydrocarbons, or other organic particles) and may interact with them in the gastrointestinal tract, leading to combined effects that are difficult to distinguish toxicologically. Therefore, future studies should also focus on the synergistic and antagonistic interactions among different particles. One of the key challenges in assessing the health risks of MPs to humans is the limited knowledge of actual human exposure levels, as most human-related data are still extrapolated from animal studies. Moreover, no threshold values causing irreversible damage to the human body have yet been established; hence, a cautious but thorough interpretation is required [157]. There is an urgent need to develop and standardize advanced analytical tools enabling effective sampling, isolation, detection, quantitative determination, and characterization of MPs, especially particles smaller than 10 µm, including nanoplastics, which have been proven to penetrate biological barriers. Current estimates of external exposure are based on limited and highly diverse data, mainly concerning larger particles (10–50 µm), together with the lack of appropriate standardization of procedures and quality control of analyses, which significantly hinders comprehensive risk assessment and distorts the overall hazard evaluation [110]. In practice, an important course of action is the development of technologies that will reduce MP emissions. Another point should be the change and strengthening of legal regulations, as well as conducting educational campaigns raising public awareness about the consequences they will face in the near future. An interdisciplinary approach combining chemical, natural, medical, and social sciences should prove crucial, contributing to an effective and responsible response to the growing threat.

## Figures and Tables

**Table 1:** Biotic degradation of polymers used in the food industry.

Polymer	Enzymes Involved/Likely Involved in the Degradation Process	Examples Microorganisms	Degradation Products
PE	MnP—manganese peroxidase (reduces tensile strength and average molecular weight) SBP—soy peroxidase (oxidizes the surface and increases hydrophobicity in combination with hydrogen peroxide), Lac—laccase (oxidizes the surface, reduces molecular weight) AlkB—alkane hydroxylases—enzymes involved in the alkane degradation pathway, play a key role in the breakdown of linear alkanes, and are currently the best-characterized enzymes involved in PE degradation within β-oxidation [42]	*Streptomyces* spp. [43], *Bacillus subtilis*, *B. licheniformis* [44], *Serratia marcescens*, *Meyerozyma guilliermondii* [45], *Enterobacter ludwigii*, *Chryseobacterium* sp., *Pseudomonas fluorescens*, *P. megaterium*, *Klebsiella pneumoniae* [41], *Aspergillus niger*, *A. nidulans*, *A.flavus*, *A. glaucus* [46], *Penicillium pinophilum* [47], *Fusarium* sp. *F. solani*, *F. oxysporum* [41]	aldehydes (e.g., formaldehyde, acetaldehyde), ketones, alcohols, carboxylic acids (formic, acetic, propionic), final carbon dioxide (CO_2_), water (H_2_O), biomass (aerobic conditions). methane (CH_4_), carbon dioxide (CO_2_), water (H_2_O), biomass (anaerobic conditions) [48]
PP	Lac—laccase (catalyzes surface oxidation) MnP—manganese peroxidase (oxidation of polymer fragments) [49]	*Alcaligenes* sp., *Pseudomonas* sp., *Vibrio* sp., *Bacillus cereus*, *Sporosarcina globispora*, *Serratia marcescens*, *Stenotrophomonas panacihum*, *Aspergillus* sp. (*niger*, *terreus*, *flavus*, *fumigatus*), *Aureobasidium pullulans*, *Chaetomium globosum*, *Paecilomyces variotii*, *Scopulariopsis brevicaulis* [41], *Engyodontium album*, *Phanerochaete chrysosporium* [34], *Lasiodiplodia theobromae* [50]	aldehydes, ketones, carboxylic acids, final carbon dioxide (CO_2_) and water, biomass under anaerobic conditions, methane (CH_4_) [49]
PS	SMO—styrene monooxygenase (converts styrene to oxide) SOI—styrene oxide isomerase, PAALDH—phenylacetaldehyde dehydrogenase, PAAH—phenylacetate hydroxylase, HPAAH—2-hydroxyphenylacetate hydroxylase, HGADO—homogentisate dioxygenase (converts styrene oxide to 4-maleylacetoacetate). SDO—styrene dioxygenase (hydroxylation of styrene in the aromatic ring, leading to the formation of cis-styrene glycol) CGDH—cis-glycol dehydrogenase, CDO—catechol 2,3-dioxygenase, HMASALDH—2-hydroxymuconic semialdehyde hydrolase, HPDEH—2-hydroxypenta-2,4-dienoate hydratase, HOA—4-hydroxy-2-oxovalerate aldolase, PDHC—pyruvate dehydrogenase complex (converts cis-styrene glycol to acetyl-CoA) [40] Oxidases and oxygenases (may participate in the cleavage of C–C bonds in the polystyrene structure) [51]	*Bacillus* sp. [37], *Pseudomonas* sp., *Micrococcus* sp., *Nocardia* sp. *Exiguobacterium* sp. [52], *Rhodococcus ruber* [53], *Curvularia* sp. [54], *Cephalosporium* sp., *Mucor* sp. [55], *Aspergillus niger*, *Trichoderma* sp., *Pullularia pullulans* [41]	styrene, styrene dimers and trimers, aromatic styrene oxidation products (toluene, ethylbenzene, etc.), oxidized styrene metabolites (styrene oxide, phenylacetaldehyde), phenylacetic acid, and organic acids that are products of the Krebs cycle. End products of biotic degradation: carbon dioxide (CO_2_), water (H_2_O), biomass and under anaerobic conditions, additionally methane (CH_4_) [56]
PET	PETase (degrades the polymer surface into smaller fragments of MHET), MHETase (mono(2-hydroxyethyl) terephthalate hydrolase) acts in cooperation with PETase and hydrolyzes MHET into terephthalic acid (TPA) and ethylene glycol (EG) [57] Cutinases, lipases [58], esterases (oxidize PET ester bonds) [59]	*Ideonella sakaiensis* [60], *Bacillus* sp. BCBT21 [61], *Bacillus subtilis* [62], *Thermobifida alba* [63], *Thermomyces lanuginosus* [58], *Priestia aryabhattai*, *Bacillus pseudomycoides*, *Bacillus pumilus* [64]	PET monomers and oligomers, TPA, protocatecholic acid, EG, glycolaldehyde, organic acids, and ultimately carbon dioxide (CO_2_), water (H_2_O), biomass [56]

**Table 2:** Methods used to monitor the degree of degradation of plastics.

Analyzed Parameter	Obtained Data	Method
Surface morphology	Changes in surface structure, color changes, formation of cracks and holes, surface roughness, and development of microbial colonies	SEM (Scanning Electron Microscopy) [52], AFM (Atomic Force Microscopy) [68]
Mechanical properties	Breaking load, tensile strength, time of elongation	UMTS (Universal Mechanical Testing System) [69]
Mass	Measured polymer weight loss determined before and after the degradation process	Gravimetric method [37,55]
Molecular weight	Change in molecular weight distribution, such as a decrease in average molecular weight, can serve as a preliminary method to assess the sites of polymer chain scission during plastic degradation	GPC (Gel Permeation Chromatography) [40,52,70]
Metabolic products	Characterization of individual intermediate metabolic products, e.g., possible oxidative cleavage products such as alcohols, esters, and acids [40]	MS (Mass Spectrometry), LC-MS (Liquid Chromatography-Mass Spectrometry) [71]
Functional groups	Measurement of chemical modifications and formation of functional groups on the surface of plastics after microbial degradation, and identification of chemical components. For example, increased infrared absorbance around 3400 cm^−1^ for hydroxyl groups [54]; bands in the 1710–1715 cm^−1^ range in infrared or the appearance of bands at 288.2 eV (XPS) for carbonyl groups (-C=O-) [40]	FTIR (Fourier Transform Infrared Spectroscopy) [54,72], XPS (X-ray Photoelectron Spectroscopy) [52], NMR (Nuclear Magnetic Resonance) [73,74]
CO_2_/CH_4_	Quantitative determination of oxygen consumption or biogas production (when the polymer is the sole carbon source for microorganisms, CO_2_ is produced under aerobic conditions, or CO_2_ and CH_4_ under anaerobic conditions). Thus, the amount of produced gases or the oxygen consumed during biodegradation can serve as an analytical parameter to assess ultimate biodegradation [40]	Respirometer, GC (Gas Chromatography) [72]

**Table 3:** Food products contaminated with MPs.

Product Type	MP Type/Content	Form	Source	References
Tap water	PVC, PE, PA, PP 4.7 × 10^3^ items/year	fragments, fibers, spherical beads	plastic pipes, coated iron pipes (leaching especially at alkaline pH), and plastic membrane filters.	[112,113]
Water in plastic bottles	PET 84%, PP 7%, PE 5% 118 ± 88 items/l	mixed particle types	packaging. MPs originating from bottles made of PET and caps made of PP. Higher amounts of MPs in carbonated water result from increased stress on the plastic material, which releases more particles	[111]
Water in glass bottles	41% PEST (PET and PES) 35% PE, 12%PA, 8%PP 50 ± 52 items/l	mixed particle types	abrasion of the soft plastic material of the bottle cap and seal, and the production processes.	[111]
Carton-packaged beverages	38% PE, 32% PEST, 26% PP 11 ± 8 items/l	mixed particle types	packaging. Coating of cartons with polyethylene film and caps treated with lubricants.	[111]
Tea bags	PE, PET 200–500 items/g	fragments, fibers,	contamination of the environment where the plants grow, the production process, drying of the raw material, plastic tea bags, and the release of MPs during brewing.	[114]
Beer	12–10^9^ fragments/L 2–79 fibers/L 2–66 pellets/L	fragments, fibers, pellets	atmosphere, emission of particles into the air (from machines, people, external air)	[115]
Milk	sulfonated polymers 6.5 ± 2.3 items/l	fibers, fragments	membrane materials in dairy processes	[116]
Salt	PP (40%), PE (33,3%) PET (7%)	fragments, fibers	contamination of brines by ocean waters	[117]
Sugar	a mixture of MPs fibers (217 ± 123/kg) fragments 32 ± 7/kg	fragments, fibers	pigment particles, encrustations, textiles, dirt	[118]
Honey	a mixture of MPs fibers (40–660/kg) fragments (0–38/kg)	fibers, fragments	particles transported by bees to the hive; introduction during honey processing	[118]
Eggs	PE 11.67 ± 3.98 items/egg	pellets	egg packaging, external natural environments, and feed	[119]
	Packaged food			
Packaged meat	XPS (extruded polystyrene) 4.0–18.7 items/kg	fibers	food packaging, production process, environmental contamination	[120]
Rice in bags	PE (present in food sample) no concentration data)	particles	food packaging (generation of a greater amount of MPs during cooking), contamination of raw material during the production stage, or the environment	[121]
Packaged cheese	LDPE, PP, PE (present in food sample), no concentration data)	particles	food packaging (refrigeration conditions), contamination during the production stage	[122]
Fish				
*Thunnus thynnus* (bluefin tuna)	PP 35% PE 20.4% 160/270 items/kg	fragments, filaments, spheroids	MPs contamination depends on how and where fish feed (predators, benthic fish, etc.) and where they occur. MPs enter fish flesh through the food web or direct exposure.	[123]
*Xiphias ladius* (swordfish)	PP 33%, PVA 28.4%, PVC 18.3% 140–270 items/kg of muscle	fragments, filaments, spheroids		[123]
*Platycephalus indicus* (flathead fish)	a mixture of MPs 18.50 ± 4.55 items/10 g fish muscle	fragments, fibers, pellets		[124]
*Sphyraena jello* (yellow barracuda)	a mixture of MPs 5.66 ± 1.69 item/10 g fisch muscle	fragments, fibers, pellets		[124]
	Shrimp			
*Penaeus semisulcatus* (green tiger prawn)	a mixture of MPs 0.36 items/g	fibers, fragments	bottom sediments containing MPs serve as a feeding ground for shrimp (benthic species)	[116,125,126]
*Penaeus indicus* (indian white prawn)	a mixture of MPs 0.178 items/g	fibers, fragments		
*Parapenaeopsis stylifera* (kadal shrimp)	a mixture of MPs 0.256 items/g	fibers, fragments		
Crabs	CP (cellophane) 34.87%, PET 23.11%, PE 16.39%, PP 13.45% 5.17 ± 4.43 items/individual	fibers, fragments, sheets, and microbeads	contamination present in water and bottom sediments, dependent on the feeding location and the feeding method	[127]
Muscles				
Mytilus edulis edible mussel	a mixture of MPs 0.36 items/g	particles	contamination present in seawater—algae, which constitute their food	[128]
Crassostrea gigas oysters	a mixture of MPs 0.47 items/g			

## Data Availability

Data sharing is not applicable to this article as no new data were created or analyzed in this study.

## Abbreviations

The following abbreviations are used in this manuscript:

PE	Polyethylene
PP	Polypropylene
PS	Polystyrene
PET	Polyethylene Terephthalate
MP	Microplastic
NP	Nanoplastics
SUP	Single-Use-Plastics
PVC	Polyvinyl Chloride
ABS	Acrylonitrile-Butadiene-Styrene
PA	Polyamide
PI	Polyimide
PMMA	Polymethyl Methacrylate
PTFE	Polytetrafluoroethylene
PVDC	Polyvinylidene chloride
LDPE	Low-Density Polyethylene
LLDPE	Linear Low-Density Polyethylene
HDPE	High-Density Polyethylene
BLDPE	Branched Low-Density Polyethylene
OECD	Organization for Economic Co-operation and Development
MFI	Melt Flow Index
HPP	Polypropylene Homopolymer
RCP	Random Copolymer
ICP	Impact Copolymer
BASF	Badische Anilin- and Soda-Fabrik
GPPS	General Purpose Polystyrene
HIPS	High Impact Polystyrene
EPS	Expandable Polystyrene
EG	Ethylene Glycol
TPA	Terephthalic Acid
DMT	Dimethyl Terephtalate
MnP	Manganese Peroxidase
SBP	Soybean Peroxidase
Lac	Laccase
AlkB	Alkane Hydroxylase
SMO	Styrene Monooxygenase
SOI	Styrene Oxide Isomerase
PAALDH	Phenylacetaldehyde Dehydrogenase
PAAH	Phenylacetate Hydroxylase
HPAAH	2-hydroxyphenylacetate Hydroxylase
HGADO	Homogentisate Dioxygenase
SDO	Styrene Dioxygenase
CGDH	Cis-Glycol Dehydrogenase
CDO	Catechol 2,3-Dioxygenase
SALDH	2-Hydroxymuconic Semialdehyde Hydrolase
HPDEH	2-Hydroxypenta-2,4-Dienoate Hydratase
HOA	4-Hydroxy-2-Oxovalerate Aldolase
PDHC	Pyruvate Dehydrogenase Complex
MHETase	Mono(2-Hydroxyetyl) Terephthalate Hydrolase
BHET	Bis(2Hydroxyethyl)Terephthalate
LiP	Lignin Peroxidase
LMEs	Lignin-Modifying Enzymes
SEM	Scanning Electron Microscopy
AFM	Atomic Force Microscopy
UMTS	Universal Mechanical Testing System
GPC	Gel Permeation Chromatography
MS	Mass Spectrometry
LC-MS	Liquid Chromatography-Mass Spectrometry
FTIR	Fourier Transform Infrared Spectroscopy
XPS	X-ray Photoelectron Spectroscopy
NMR	Nuclear Magnetic Resonance
GC	Gas Chromatography
TCA	Tricarboxylic acid
PHA	Polyhydroxyalkanoates
PAHs	Polycyclic Aromatic Hydrocarbons
HCFC-22	Hydrochlorofluorocarbon-22
Tg	Transition Temperature
COFs	Covalent Organic Frameworks
MOFs	Metal–Organic Frameworks
GPGP	Great Pacific Garbage Patch
DDT	Dichlorodiphenyltrichloroethane
HOCs	Halogenated Organic Compounds
PES	Polyethylene Succinate
PEST	PET and PES
XPS	Extruded Polystyrene
PVA	Polyvinyl Alcohol
CP	Cellophane
SOD	Superoxide Dismutase
MDA	Malondialdehyde
ROS	Reactive Oxygen Species
ALT	Alanine Aminotransferase
AST	Aspartate Aminotransferase
LDH	Lactate Dehydrogenase
IL-1β	Interleukin-1 beta
TNF-α	Tumor Necrosis Factor alpha
CYP450	Cytochrome P450 family isoenzymes
PI3K/Akt	Phosphoinositide 3-Kinase/Protein Kinase B
PLA	Polylactic acid
